# Oceanographic variation influences spatial genomic structure in the sea scallop, *Placopecten magellanicus*

**DOI:** 10.1002/ece3.3846

**Published:** 2018-02-11

**Authors:** Mallory Van Wyngaarden, Paul V. R. Snelgrove, Claudio DiBacco, Lorraine C. Hamilton, Naiara Rodríguez‐Ezpeleta, Luyao Zhan, Robert G. Beiko, Ian R. Bradbury

**Affiliations:** ^1^ Department of Biology Memorial University of Newfoundland St. John's NL Canada; ^2^ Department of Ocean Sciences Memorial University of Newfoundland St. John's NL Canada; ^3^ Bedford Institute of Oceanography Dartmouth NS Canada; ^4^ Aquatic Biotechnology Lab Bedford Institute of Oceanography Dartmouth NS Canada; ^5^ Marine Research Division AZTI Sukarrieta Bizkaia Spain; ^6^ Faculty of Computer Science Dalhousie University Halifax NS Canada; ^7^ Fisheries and Oceans Canada Northwest Atlantic Fisheries Centre St. John's NL Canada

**Keywords:** adaptation, outlier loci, population genomics, RAD‐seq, sea scallop, single‐nucleotide polymorphism

## Abstract

Environmental factors can influence diversity and population structure in marine species and accurate understanding of this influence can both improve fisheries management and help predict responses to environmental change. We used 7163 SNPs derived from restriction site‐associated DNA sequencing genotyped in 245 individuals of the economically important sea scallop, *Placopecten magellanicus*, to evaluate the correlations between oceanographic variation and a previously identified latitudinal genomic cline. Sea scallops span a broad latitudinal area (>10 degrees), and we hypothesized that climatic variation significantly drives clinal trends in allele frequency. Using a large environmental dataset, including temperature, salinity, chlorophyll a, and nutrient concentrations, we identified a suite of SNPs (285–621, depending on analysis and environmental dataset) potentially under selection through correlations with environmental variation. Principal components analysis of different outlier SNPs and environmental datasets revealed similar northern and southern clusters, with significant associations between the first axes of each (*R* ^2^_adj_ = .66–.79). Multivariate redundancy analysis of outlier SNPs and the environmental principal components indicated that environmental factors explained more than 32% of the variance. Similarly, multiple linear regressions and random‐forest analysis identified winter average and minimum ocean temperatures as significant parameters in the link between genetic and environmental variation. This work indicates that oceanographic variation is associated with the observed genomic cline in this species and that seasonal periods of extreme cold may restrict gene flow along a latitudinal gradient in this marine benthic bivalve. Incorporating this finding into management may improve accuracy of management strategies and future predictions.

## 1 INTRODUCTION

The application of population genomic‐based approaches to the study of marine population structure has revealed increasingly higher levels of genetic differentiation and population structure than previously recognized in multiple marine species (e.g., Benestan et al., 2015; Bradbury et al., 2013; Corander, Majander, Cheng, & Merila, 2013; Milano et al., 2014; Moura et al., 2014). Recent observations of fine‐scale differentiation are changing our view of marine connectivity and marine population dynamics (Hauser & Carvalho, 2008). Limited dispersal may contribute to fine‐scale population differentiation, but given large populations and large environmental gradients, selection may also contribute significantly to genetic differentiation among marine populations (Hauser & Carvalho, 2008). As such, studies supporting a role for selection in regulating marine connectivity continue to accumulate (Bradbury et al., 2010; Clarke, Munch, Thorrold, & Conover, 2010; Limborg et al., 2012; Milano et al., 2014; Sjöqvist, Godhe, Jonsson, Sundqvist, & Kremp, 2015; Van Wyngaarden et al., 2017). Researchers increasingly recognize the important role of selection in population connectivity, particularly for economically important species, because an accurate understanding of population structure and environmental influences can contribute to the identification of conservation units and allow prediction of a species' response to climate change (Allendorf, Hohenlohe, & Luikart, 2010; Conover, Clarke, Munch, & Wagner, 2006; Sale et al., 2005).

Genomic studies increasingly highlight a role for selection in regulating marine population structure (Berg et al., 2015; Bradbury et al., 2010, 2014; Gagnaire et al., 2015; Gaither et al., 2015; Hellberg, 2009), and loci identified as putatively under selection repeatedly reflect small‐scale genetic differentiation in multiple marine species (Bradbury et al., 2010; De Wit & Palumbi, 2013; Lamichhaney et al., 2012). Additionally, marine landscape genomic studies combining traditional landscape approaches with large genomic datasets have identified significant associations between climate and genetic structure (genetic–environmental associations) in numerous marine and anadromous species, including Atlantic herring (*Clupea harengus*) (Limborg et al., 2012), Atlantic cod (*Gadus morhua*) (Berg et al., 2015; Bradbury et al., 2010), purple sea urchin (*Strongylocentrotus purpuratus*) (Pespeni & Palumbi, 2013), Atlantic salmon (*Salmo salar*) (Bradbury et al., 2014), European hake (*Merluccius merluccius*) (Milano et al., 2014), and Chinook salmon (*Oncorhynchus tshawytscha*) (Hecht, Matala, Hess, & Narum, 2015). Although historically most marine population genomic studies have focused on fish species, work on other taxa, including invertebrates, is increasing (e.g., Benestan et al., 2015; Pespeni & Palumbi, 2013). The pervasiveness of genetic–environmental associations across taxa and life histories supports the hypothesis that environmentally associated selection may structure marine populations.

**Figure 1:**
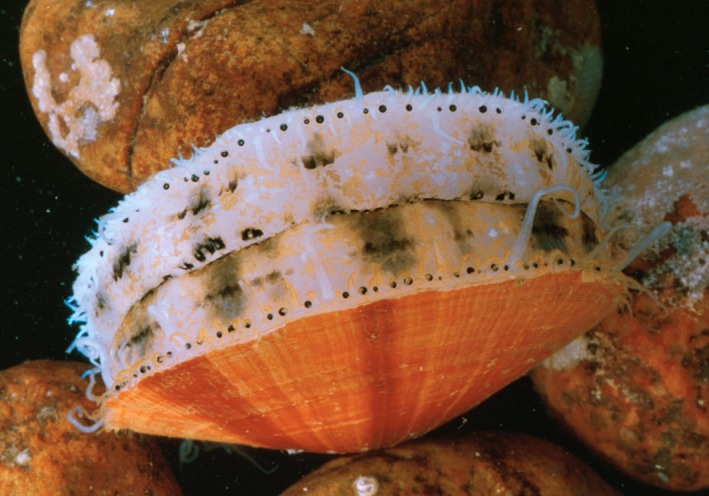
*Placopecten magellanicus*. By Dann Blackwood, USGS [Public domain], via Wikimedia Commons

The sea scallop, *Placopecten magellanicus* (Gmelin) (Figure 1), is an economically important benthic marine bivalve with a range that extends from North Carolina, USA, to Newfoundland, Canada (Posgay, 1957). The scallop fishery in both the United States and Canada extends back over 100 years and is one of the most economically important fisheries on the east coast of North America (DFO 2016; Naidu & Robert, 2006; NOAA 2016). The sessile adult scallops live in isolated beds up to 300 m deep and undergo broadcast spawning. Juvenile scallops have a planktonic period of development of up to 40 days, which is conducive with the potential for long distance dispersal among populations (Davies, Gentleman, DiBacco, & Johnson, 2014; Tian et al., 2009). The scallop range spans a vast latitudinal area where the cold Labrador Current meets the warm Gulf Stream. This convergence leads to large gradients in ocean temperature and other environmental factors experienced by different scallop populations, all of which may be influenced by the oceanographic properties of major currents and storm‐related mixing along coastal areas (Townsend, Thomas, Mayer, Thomas, & Quinlan, 2006). Several oceanographic barriers (such as current fronts) along the range may also influence larval dispersal and survival among populations (Townsend et al., 2006).

Previous genetic work on the sea scallop detected significant but weak population structure among geographic locations off eastern Northern America using microsatellites, AFLPs, and SNPs (Kenchington, Patwary, Zouros, & Bird, 2006; Owen & Rawson, 2013; Van Wyngaarden et al., 2017). Recently, Van Wyngaarden et al. (2017) resolved significant spatial structuring in sea scallops visible primarily in outlier loci detected using *F*_ST_‐based outlier detection methods and genomewide RAD‐seq (restriction site‐associated DNA sequencing)‐derived SNPs. Taken together, the results from these studies suggest that both limited dispersal and selection associated with local adaptation across the species range may spatially structure scallop populations, despite high potential for gene flow. Here, we build directly on existing studies and known population structure to identify environmental variables that may contribute to non‐neutral divergence among sea scallop populations. Using the RAD‐seq‐derived SNPs identified in Van Wyngaarden et al. (2017), we focus solely on environmental association‐based outlier tests to identify loci potentially under selection and directly compare our results to existing work and the results of previous outlier tests.

Considering the unique oceanographic features, the large latitudinal range, and previously identified clinal population structure along the range of the sea scallop, we hypothesize that directional selection and local adaptation drive sea scallop population structure and that ocean temperature likely contributes significantly to adaptation of the species to its local environment. Our specific objectives are to: (1) explore spatial variation in environmental variables across the range of the sea scallop, (2) use environmental correlation‐based outlier detection methods to pinpoint potential targets of environment‐based selection across the genome of the sea scallop, and (3) identify potentially important environmental drivers of population structure and adaptation in scallops. This work represents one of the first population genomic studies on a bivalve species or on an invertebrate species with a planktonic juvenile and sessile adult life stage. This work also incorporates both environmental association‐based outlier detection and nonlinear random‐forest analysis (Breiman, 2001), a machine‐learning strategy only recently applied to genomic analysis that can help to account for interaction and covariation between variables (Brieuc, Ono, Drinan, & Naish, 2015). We extend a previous study identifying latitudinal clinal trends in allele frequency across the range using 7163 RAD‐seq‐derived SNPs (Van Wyngaarden et al., 2017) and identify environmental associations and possible mechanisms.

## 2 METHODS

### 2.1 Sample collection and RAD‐seq

**Figure 2:**
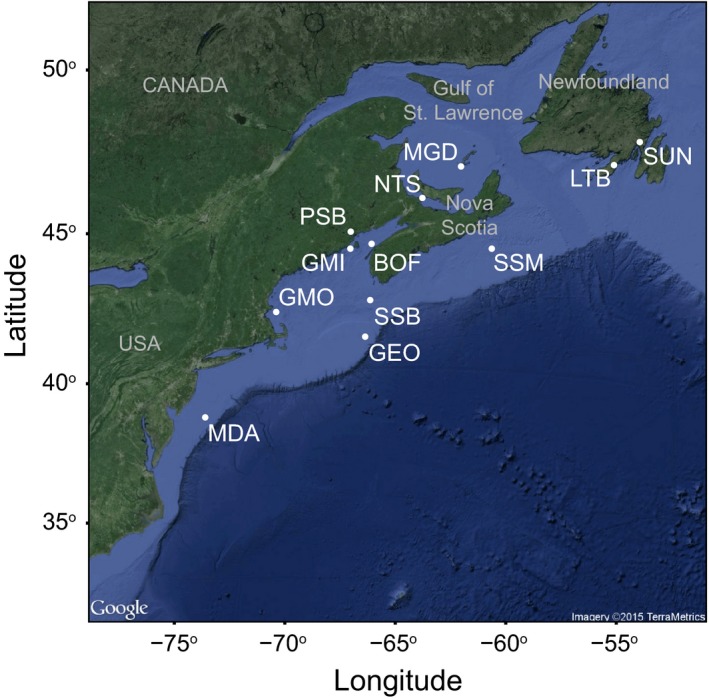
Map of sea scallop collection locations from the Northwest Atlantic. Site MDA (Mid‐Atlantic Bight) represents the middle of several nearby collection locations grouped as one population

Sample collection and RAD‐seq follow Van Wyngaarden et al. (2017). Using divers or bottom trawls, 252 adult scallops were collected from a total of 12 locations across the entire range of the species between 2011 and 2013 (Table 1, Figure 2). A minimum of 12 scallops were collected per population (mean ± *SD*, 20.4 ± 2.8 scallops).

**Table 1:** Site name, site code, coordinates, and the number of sequenced *P. magellanicus* from each of 12 collection sites in the Northwest Atlantic Ocean

Site name	Site code	Latitude	Longitude	Number of scallops used in analysis
Sunnyside, NL	SUN	47.82	−53.87	20
Little Bay, NL	LTB	47.15	−55.10	21
Magdalen Islands	MGD	47.11	−62.02	21
Northumberland Strait	NTS	46.13	−63.77	22
Passamaquoddy Bay	PSB	45.06	−67.02	12
Bay of Fundy	BOF	44.68	−66.07	22
Scotian Shelf ‐ Middle	SSM	44.52	−60.64	19
Gulf of Maine Inshore	GMI	44.52	−67.03	20
Browns Bank	SSB	42.84	−66.14	22
Gulf of Maine Offshore	GMO	42.44	−70.39	22
George's Bank	GEO	41.61	−66.36	22
Mid Atlantic Bight^a^	MDA	38.82	−73.60	22

a Several neighbouring sites sampled as one location

Muscle tissue samples were collected and preserved in AllProtect (Qiagen) or 80% ethanol. DNA extraction and RAD‐seq library preparation were performed at the Aquatic Biotechnology Lab, Bedford Institute of Oceanography in Dartmouth, Nova Scotia. RAD‐seq libraries were prepared using *Sbf1* as described by Etter, Preston, Bassham, Cresko, and Johnson (2011) (see also Etter, Bassham, Hohenlohe, Johnson, and Cresko, 2011) with modifications. Each library was created using 22 individuals from each sampling location (or 20 individuals for SUN) with a unique in‐line barcode in the P1 adapter for each individual. The P1 adapter barcodes were 6 bp in length for all populations except for SSB, GEO, and SUN where the barcodes ranged from 5 bp to 9 bp. The barcodes for SSB, GEO, and SUN were chosen to ensure equal distribution of all nucleotides at each base position and to maximize the edit distance (Faircloth & Glenn, 2012). Based on edit tags analysis (Faircloth & Glenn, 2012), the variable length barcodes edit distance ranged from two to eight (modal edit distance was six). Sequencing was performed at the McGill University and Génome Québec Innovation Centre, Montréal, Canada, using a HiSeq 2000 (Illumina) as 100‐bp paired‐end sequences with one library per lane.

SNPs were detected using the de novo pipeline in STACKS v.0.9999 (Catchen, Amores, Hohenlohe, Cresko, & Postlethwait, 2011). Loci were assembled using *ustacks,* requiring a minimum depth of coverage for a stack (m) of five and allowing four maximum nucleotide mismatches (M) between stacks. The catalog of loci was assembled using *cstacks* allowing a distance between loci in the catalog (n) of six. The final dataset was filtered using PLINK v.1.07 (Purcell, 2009; Purcell et al., 2007) to include only RADtags present in 75% of individuals in SNP discovery and calling; all SNPs included in the analysis were present in 75% of individuals with a minor allele frequency (MAF) greater than 5%. Furthermore, we excluded individuals with more than 20% missing loci from the analysis. Loci were filtered for Hardy–Weinberg equilibrium using the program GENEPOP v.4 (Rousset, 2008), excluding loci out of equilibrium in six or more populations from the analysis (<0.7% of all loci).

### 2.2 Environmental data collection and processing

We amalgamated environmental data from several databases; from Fisheries and Oceans Canada: Climate (Gregory, 2004) (years 1970–2013), BioChem (Devine et al., 2014; DFO 2014) (years 2009–2014), and AZMP (DFO 2015), and from the National Oceanographic and Atmospheric Administration in the United States of America (NOAA, years 1990–2010), and the MODIS satellite database (NASA Goddard Space Flight Center Ocean Ecology Laboratory 2014) (years 2002–2013). Data were averaged over multiple years available to remove the signatures of short‐term variation in the marine environment. Available physical and chemical variables included water temperature, salinity, sigma‐t (a measure of water density related to temperature and salinity), chlorophyll a (a measure of primary productivity), and concentrations of SiO_4_^4−^, NO_3_^−^, NO_2_^−^, and PO_4_^3−^ (nutrients required for many marine primary producers).

We averaged data from all data sources within a bounding box of one square degree around each sample site to create site‐specific averages for each data type used in the analysis. Data were separated into surface and depth values based on the collection depth for each sampling location. Surface values encompassed depths between zero and 20 m except for collection sites less than 20 m depth, where 10 m was used as the surface cutoff. We averaged values from a cutoff approximately 10 m above a given collection depth to the collection depth for depth‐profiled variables. In cases where multiple sample collection depths were provided, depth cutoff parameters were altered to include the entire range of collection depths (Table 2).

**Table 2:** Depth and surface ranges and bounding box coordinates used to select environmental data from databases for each of 12 *P. magellanicus* collection sites in the Northwest Atlantic Ocean

Site name	Site code	Depth range (m)	Surface range (m)	Bounding box top left		Bounding box bottom right	
				Latitude	Longitude	Latitude	Longitude
Sunnyside, NL	SUN	−10 to −20	0 to −10	48.82	−54.87	46.82	−52.20
Little Bay, NL	LTB	−30 to −40	0 to −20	48.15	−56.10	46.15	−54.10
Magdalen Islands	MGD	−35 to −45	0 to −20	48.11	−63.02	46.11	−61.02
Northumberland Strait	NTS	−15 to −25	0 to −10	47.13	−64.77	45.13	−62.77
Passamaquoddy Bay	PSB	−20 to −30	0 to −20	46.06	−68.02	44.06	−66.02
Bay of Fundy	BOF	−30 to −140	0 to −20	45.68	−67.07	43.68	−65.07
Scotian Shelf ‐ Middle	SSM	−35 to −50	0 to −20	45.52	−61.64	43.52	−59.64
Gulf of Maine Inshore	GMI	−60 to −80	0 to −20	45.52	−68.03	43.52	−66.03
Browns Bank	SSB	−50 to −125	0 to −20	43.84	−67.14	41.84	−65.14
Gulf of Maine Offshore	GMO	−70 to −90	0 to −20	43.44	−71.39	41.44	−69.39
George's Bank	GEO	−50 to −100	0 to −20	42.61	−67.36	40.61	−65.36
Mid Atlantic Bight	MDA	−70 to −90	0 to −20	39.82	−74.60	37.82	−72.60

Data validation and preparation were completed using R (R Core Development Team 2012). To address natural seasonal variation in the data, we calculated *z*‐scores for each variable for each sample site per month and removed outliers where necessary. Variables with missing data for more than six sites were removed from subsequent analyses (29 variables in total were removed). For the remaining variables with missing data, we used single imputation using neighboring sites to estimate missing values (sites arranged by latitude, averaging sites directly north and south of the missing site). Following outlier removal and imputation, we standardized data to zero mean and unit variance by subtracting the mean and dividing by the standard deviation. We then identified site‐specific maximum and minimum values as well as seasonal averages for each variable, basing seasons largely on equinoxes. Winter included January, February, and March; spring included April, May, and June; summer included July, August, and September; and autumn included October, November, and December. The final dataset contained 90 variables spanning all available data types (hereby referred to as AllEnv). The 29 variables removed due to missing data translated to only six removed final variables following minimum, maximum, and seasonal calculations. We repeated all analyses using only the temperature, salinity, and chlorophyll a variables (*n* = 36 variables, henceforth CST), given that we expected these to be the most likely to associate with selection; they not only provide evidence of food availability but also characterize water mass properties that can affect all trophic levels.

### 2.3 Detection of outlier loci

We used two separate methods to detect outlier loci using both environmental datasets (four tests in total). The first method used a Bayesian framework implemented in the program BAYENV2 (Coop, Witonsky, Di Rienzo, & Pritchard, 2010; Guenther & Coop, 2013). This method calculates a set of “standardized allele frequencies” that controls for population history and structure when detecting loci whose allele frequencies show significant associations with environmental variation. This method then calculates a Bayes factor (BF), which measures the weight of evidence for a model in which the environmental variable affects the allele frequency of a locus versus a null model with no environmental variable effect. To calculate the “standardized allele frequencies,” we randomly selected 700 loci (9.8% of total loci) identified as neutral (not under selection) in Van Wyngaarden et al. (2017). The null model correlation matrix was estimated from these loci in three repetitions of 100000 iterations. We visually compared correlation matrices from the final iterations of each run to each other and to an *F*_ST_ matrix of the neutral loci and determined there were no differences in the major patterns of the matrices. The final matrix from the first run was selected as the neutral matrix for use in further analysis. The final analysis detected locus‐specific deviations from the “standardized allele frequencies” using 100,000 iterations. BFs were calculated at every locus for each environmental variable separately. To assess the significance of each BF (and the likelihood of classifying a locus as an outlier), we created five bins of loci based on the global minor allele frequency, as recommended in Coop et al. (2010) and implemented in Hancock et al. (2010) (Table 3). We selected loci with BFs in the top 5% of the range of BFs for each bin as outliers.

**Table 3:** Number of loci and minor allele frequency (MAF) range included in each of five bins (based on global minor allele frequency) used by the program BAYENV2 to detect loci potentially under selection among 12 populations of *Placopecten magellanicus*

Bin	Number of loci	MAF range
A	3,566	0.05–0.139
B	1,390	0.14–0.229
C	908	0.23–0.319
D	691	0.32–0.409
E	608	0.41–0.5

Latent factor mixed models (LFMMs) as described in Frichot, Schoville, Bouchard, and François (2013) were implemented as the second method of outlier detection in the R package *LEA* (Frichot & François, 2015). This method uses latent factors in a linear mixed model to control for population structure (the number of genetic clusters within a group of populations, K) while detecting correlations between environmental and genetic variation. Previous analysis using the program STRUCTURE v.2.2.4 (Pritchard, Stephens, & Donnelly, 2000) detected two genetic clusters (*K* = 2) (Van Wyngaarden et al., 2017), and the genomic inflation factor analysis (GIF) in *LEA* corroborated this result. The models were run for three repetitions, with a burn‐in of 5,000 followed by 15,000 iterations. We combined *Z*‐scores from the three repetitions using the median, calculated adjusted *p*‐values to correct for multiple testing, and produced a list of candidate outlier loci for each environmental variable (FDR = 0.05) following Frichot, Schoville, de Villemereuil, Gaggiotti, and Francois (2015). To ensure we included any loci potentially under selection, for AllEnv and CST separately, we combined the list of detected outliers from both BAYENV2 and LFMM to create two final outlier lists (AllEnvOutlier for AllEnv and CSTOutlier for CST).

### 2.4 Environmental factors that influence genetic variation

We conducted principal components analysis (PCA) using the AllEnvOutlier and CSTOutlier loci using the R package *adegenet* (Jombart, 2008) to examine population structure among the sampled populations at outlier loci. To examine the relationship between environmental and genetic variation among our collection sites, we calculated population‐specific allele frequencies for AllEnvOutlier and CSTOutlier using the R package *gstudio* (Dyer, 2014). Next, we ran PCA on population‐specific allele frequencies for AllEnvOutlier and CSTOutlier (AllEnvOutlierPCA and CSTOutlierPCA), and the population‐specific environmental data in AllEnv and CST (AllEnvPCA and CSTPCA) using the R package *adegenet*. Linear regression was then performed between the first principal component (PC) from AllEnvOutlierPCA (AllEnvOutlierPC1) and the first PC from the PCA on AllEnv (AllEnvPC1) as well as the first PC from CSTOutlierPCA (CSTOutlierPC1) and the first PC from the PCA on CST (CSTPC1).

We then conducted redundancy analysis (RDA), a multivariate canonical correlation analysis, using the R package *vegan* (Oksanen et al., 2015) on population‐specific allele frequencies for AllEnvOutlier and CSTOutlier and selected PCs from AllEnvPCA and CSTPCA, respectively. This analysis allowed us to determine which environmental variables used as explanatory variables in the RDA best explain the genetic population structure. Each PC that explained more than 5% of the total explainable variance in the AllEnvPCA (five axes) and CSTPCA (four axes) was selected as an explanatory variable. Backward stepwise variable selection using 1,000 or 10,000 iterations selected the most valuable environmental PCs within the model. To determine the proportion of model variation attributable to climate versus geographic distance between populations versus combined effects, we next performed partial RDA (pRDA), conditioning the genetic matrix on the distances from the furthest north population (SUN) along a one‐dimensional transect that included all populations [estimated using GOOGLE EARTH (2013)].

Multiple linear regressions quantified the direction and magnitude of the effect of environmental variables on genetic variation. We used results from RDA to select environmental variables used in the analyses. After examining weightings of the environmental variables on the important PCs selected during RDA, we selected the five most highly weighted variables from each PC for use as explanatory variables in linear mixed models. Based on results from the initial linear mixed models (see Section 2.1), we generated models focusing on measurements of water temperature at surface and at depth (Table 4). For each response variable (AllEnvOutlierPC1 and CSTOutlierPC1), we fitted a global multiple regression model with all selected environmental variables. We then used the R package *MuMIn* (Barton, 2014) to run all possible configurations of the global model and pinpointed the best model fits with AIC_c_ model selection. We examined cumulative AIC_c_ model weights to rank each parameter in order of importance and estimated coefficients for each environmental parameter using model averaging (Arnold, 2010).

**Table 4:** Data included in all multiple linear regression models used to determine the direction and magnitude of the effect of environmental variables on genetic variation among 12 populations of *Placopecten magellanicus*

Method of variable selection	Variables included	Response variable
Most highly weighted variables from AllEnvPCs selected by RDA	Deep average autumn salinity	AllEnvOutlierPC1
	Deep minimum SiO_4_	
	Surface average autumn salinity	
	Surface average winter temperature	
	Surface minimum temperature	
Most highly weighted variables from CSTPCs selected by RDA	Deep average winter temperature	CSTOutlierPC1
	Deep average minimum temperature	
	Surface average winter temperature	
	Deep maximum salinity	
	Deep average autumn salinity	
	Surface maximum chlorophyll *A*	
	Surface average spring chlorophyll *A*	
	Surface average summer chlorophyll *A*	
	Deep minimum chlorophyll *A*	
	Surface minimum chlorophyll *A*	
Temperature variables selected following the results of initial linear mixed models	Deep average autumn temperature	AllEnvOutlierPC1, CSTOutlierPC1
	Deep average spring temperature	
	Deep average summer temperature	
	Deep average winter temperature	
	Surface average autumn temperature	
	Surface average spring temperature	
	Surface average summer temperature	
	Surface average winter temperature	

We also used nonlinear random‐forest (RF) analysis to identify important environmental variables and then compared key drivers with those identified using multiple linear regressions. One key attribute of RF is the automatic computation of variable importance, which allows us to determine which environmental variables influence population structure. Additionally, RF considers interaction between predictor variables and may manage the covariation among environmental variables more effectively than the multiple linear regression approach (Brieuc et al., 2015). This ensemble approach benefits from growing a large group of decision trees to improve overall performance.

RF cannot tolerate missing data, so we used a method based on weighted k nearest neighbors (KNN) called KNNcatImpute (Schwender, 2012) to impute the missing genotypes in our genetic SNP data using the *scrime* package in R (Schwender & Fritsch, 2013). After imputation, the individual genotypes at each outlier SNP were transformed to categorical data. SNPs are a biallelic genetic marker and only two alleles and three types of genotypes can be present at each SNP; the built RF is thus a three‐class classification model. Environmental variables were used as predictors of individual genotypes at each outlier SNP using 1,001 trees.

We used permutation importance, the variable importance function built in RF, to rank the relative roles of environmental variables in influencing population structure. To obtain a reliable estimation of variable importance, we applied 10‐fold cross‐validation, dividing the entire dataset into 10 subsets. Nine subsets trained the RF model, and the other subset was used for validation; this process was repeated 10 times for each SNP genotype. In each of the 10 runs, we calculated a permutation importance array for all environmental variables. Noting that importance values can be negative, we computed the exponential values of the importance array and averaged each importance value over the total importance sum of all environmental variables to generate an importance proportion array. The importance proportions were averaged over the 10 runs to determine average importance proportions. For each SNP genotype output (621 for AllEnv and 285 for CST), an RF model was built to calculate an array of permutation importance proportions for all environmental variables. We calculated the overall average importance proportion for each environmental variable over all loci. All RF analyses were performed using *randomForest* package in R (Liaw & Wiener, 2002).

### 2.5 Gene ontology

We performed gene ontology (GO) analysis on AllEnvOutlier and CSTOutlier in the program Blast2GO (Conesa et al., 2005) using the program default parameters and InterProScan to improve GO annotation quality.

## 3 RESULTS

### 3.1 Sample collection and RAD‐seq

Following filtering and quality control steps, we included 245 individual scallop samples in our analysis (97.2% of sequenced individuals), 19672 RADtags (14.9% of initial RADtags), and 7216 SNPs (4.2% of initial SNPs) (Table 5). Read count per individual per RADtag averaged 56.12 ± 46.64 (mean ± *SD*). Final filtering required SNPs to be present in >95% of individuals with a MAF >5% and removed individuals with >20% missing data. The final dataset included 245 individual scallop samples and 7163 SNPs. Average pairwise *r* ^2^ values indicating linkage disequilibrium remained low overall (0.0044 ± 0.0098, mean ± *SD*) (Van Wyngaarden et al., 2017).

**Table 5:** Number of *Placopecten magellanicus* individuals and number of SNP loci included in initial RAD‐sequencing and final analysis following quality control

Parameter	Value
Individuals sequenced	252
Individuals following QC	245 (97.2% of Individuals sequenced)
Initial RAD tags	131,897
RAD tags following QC	19,672 (14.9% of initial RAD tags)
Initial SNPs	173,482
SNPs following QC	7,216 (4.2% of Initial SNPs)
SNPs in HWE	7,163 (99.3% of SNPs following QC)

### 3.2 Detection of outlier loci

The neutral matrices calculated to generate “standardized allele frequencies” for BAYENV2 varied little within runs and when compared to the *F*_ST_ matrix calculated for the neutral loci; we therefore chose a single matrix for further calculations with BAYENV2. LFMM identified *K* = 2 as the most supported number of clusters (and thus latent factors) using GIF analysis, with values of 0.85 for AllEnv and 0.83 for CST. According to Frichot and François (2015), *p*‐values in this analysis calibrate correctly when GIF approaches one.

Overall, LFMM identified more loci potentially under selection than BAYENV2. Combining the results from both programs, AllEnv identified 621 loci (8.7% of all loci) as under selection, whereas CST identified 285 loci (4.0% of all loci) as under selection; 250 loci were shared between the two datasets (Table 6). Using AllEnv, BAYENV2 detected 128 loci as putatively under selection (1.8% of total loci), whereas LFMM detected 511 (7.1% of total loci). Only 18 loci were common to both the BAYENV2 and LFMM sets. Using CST, BAYENV2 detected 72 loci (1.0% of total loci), whereas LFMM detected 218 (3.0% of total loci), with only five loci shared between the two methods. Within the BAYENV2 results, the AllEnv outlier list and CST outlier list shared 37 loci (Table S1). The LFMM analysis of AllEnv and CST overlapped completely in loci identified (Table S2). We also compared our combined outlier lists to the *F*_ST_‐based outliers reported in Van Wyngaarden et al. (2017) (112 outliers). The combined AllEnv outlier list and the *F*_ST_‐based list shared 53 loci, the combined CST outlier list and the *F*_ST_‐based list shared 35 loci, and all three lists shared 28 loci (Table S3).

**Table 6:** (A) Matrix of the number of outlier loci detected in *P. magellanicus* out of 7163 total loci by the methods BAYENV2 and LFMM using two environmental datasets, AllEnv and CST. The number of loci shared between different environmental datasets and programs are italicized. (B) Combined total number of loci detected from two methods, BAYENV2 and LFMM, using two environmental datasets, AllEnv and CST. The number of loci shared between the different environmental datasets is italicized

(A)	BAYENV2		LFMM	
	AllEnv	CST	AllEnv	CST
BAYENV2				
AllEnv	128	*37*	*18*	–
CST	–	72	–	*5*
LFMM				
AllEnv	–	–	511	*218*
CST	–	–	–	218

(B)	AllEnv	CST
AllEnv	621	*250*
CST	–	285

### 3.3 Patterns of genetic and environmental variation

**Figure 3:**
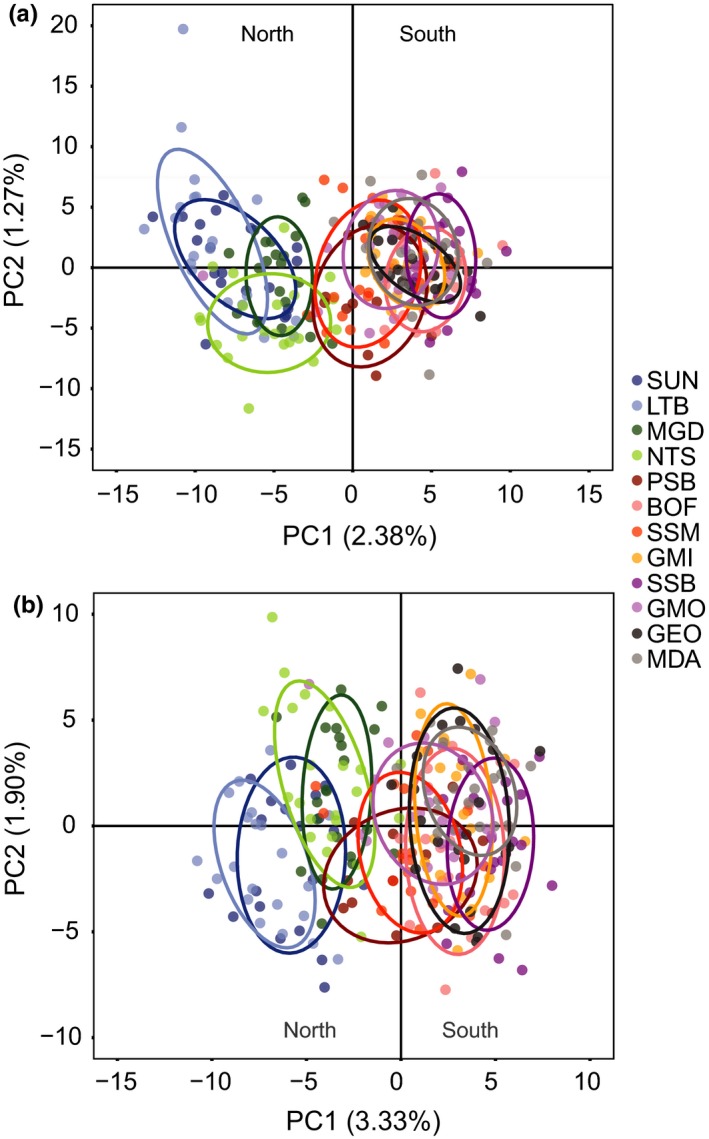
Principal components analysis plots for loci detected as potentially under selection through environmental correlation with (a) AllEnv (90 environmental variables, *n* = 621 loci), (b) CST (36 environmental variables, *n* = 285 loci) in 12 populations of *Placopecten magellanicus*

PCA of all individuals and sets of outlier loci detected using AllEnv and CST both split north and south populations along the first PC, separating the populations into two clusters as seen in the BAYENV2 results (Figure 3). Using AllEnvOutlier, the first PC explained 2.38% of the total explainable variance in the model, and using CSTOutlier, the first PC explained 3.33% of the total explainable variance. The PCA on the population‐specific allele frequencies for AllEnvOutlier and CSTOutlier (not shown) produced a similar clustering pattern; however, the first PC explained much more variance, with AllEnvOutlierPC1 explaining 26.47% of the total model variance and CSTOutlierPC1 explaining 31.93% of the total model variance.

**Figure 4:**
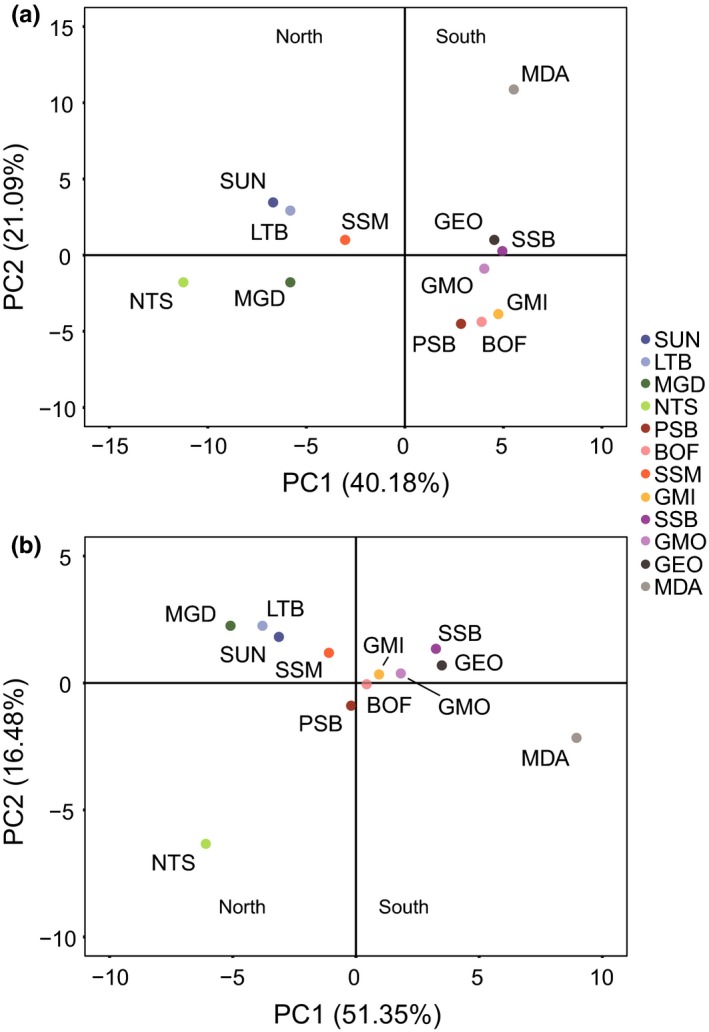
Principal components analysis plots for environmental variables used in the detection of potentially adaptive signals among 12 populations of *Placopecten magellanicus*. Environmental variables were separated into two datasets, (a) AllEnv (90 environmental variables) and (b) CST (36 environmental variables)

**Figure 5:**
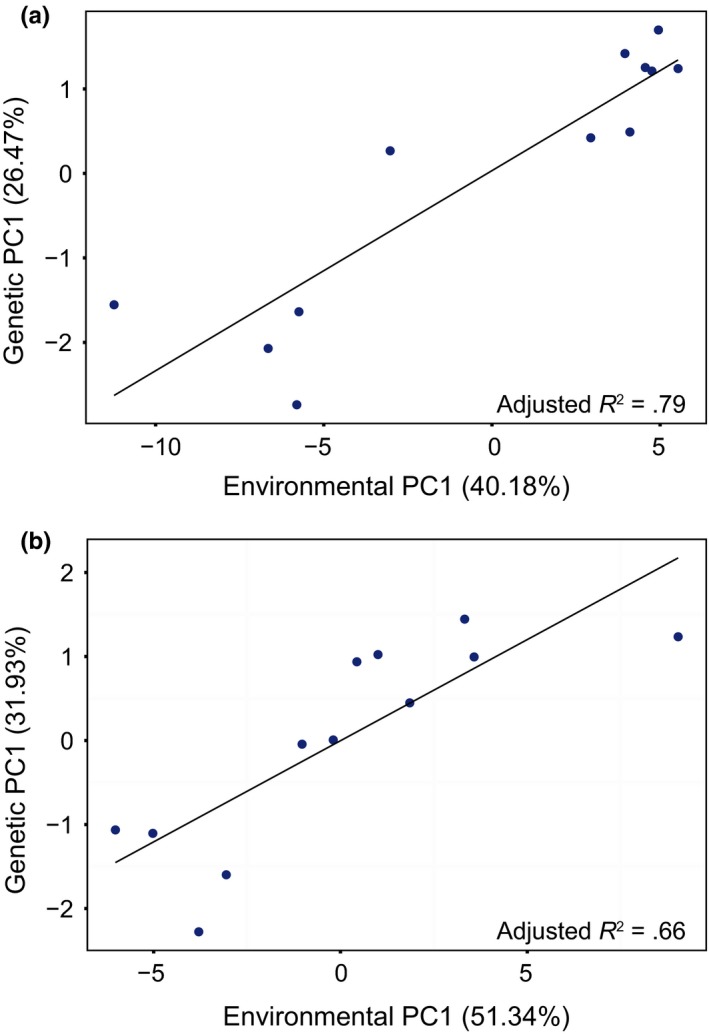
Linear regressions between the first principal component of PCA on population‐specific allele frequencies (Genetic PC1) and population‐specific environmental parameter values (Environmental PC1) for 12 populations of *Placopecten magellanicus* for (a) AllEnv (90 environmental variables, 621 loci), and (b) CST (36 environmental variables, 285 loci)

**Figure 6:**
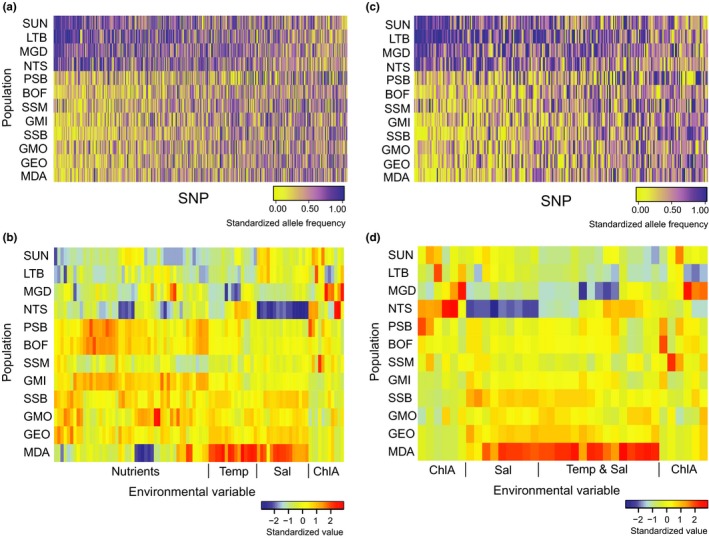
Heat map of (a) standardized major allele frequencies (AllEnvOutlier, 621 loci), (b) standardized environmental variable value (AllEnv, 90 variables), (c) standardized major allele frequencies (CSTOutlier, 285 loci), and (d) standardized environmental variable value (CST, 36 variables) for 12 populations of *Placopecten magellanicus*. Loci in (a) were selected as potentially under selection through correlation with environmental variables in (b). Loci in (c) were selected as potentially under selection through correlation with environmental variables in (d). SNPs in (a) and (c) are arrange in order of strongest to weakest differentiation pattern with major alleles based on SUN as a reference population. Variables in (b) and (d) are automatically grouped by similarity, and the main components of each group are listed below the plot

The environmental data produced the same pattern of north–south population clustering for both datasets (AllEnv and CST, Figure 4). However, these PCAs further separated the southernmost population, Mid‐Atlantic Bight (MDA), along the second PC. The first PC of the environmental data explained much more variance than in the genetic models, with AllEnvPC1 explaining 40.18% of the total model variance and CSTPC1 explaining 51.35%. Linear regressions between genetic and environmental data (i.e., AllEnvOutlierPC1 and AllEnvPC1 as well as CSTOutlierPC1 and CSTPC1) showed a strong and significant relationship (Figure 5), with adjusted *R* ^2^ values of .79 for AllEnv and .66 for CST, further indicating similar spatial patterns in genetic and environmental variation among our sample sites. The north–south population split can be seen in heat maps of standardized major allele frequency and standardized values for environmental variables in AllEnv and AllEnvOutlier and CST and CSTOutlier (Figure 6). In Figure 6b,d, lower standardized values can be seen in the four furthest north populations, and in Figure 6a,c, a similar split can be seen in standardized major allele frequencies, although only for a subset of alleles showing the strong clinal pattern driving the north–south split.

### 3.4 Environmental factors that influence genetic variation

**Figure 7:**
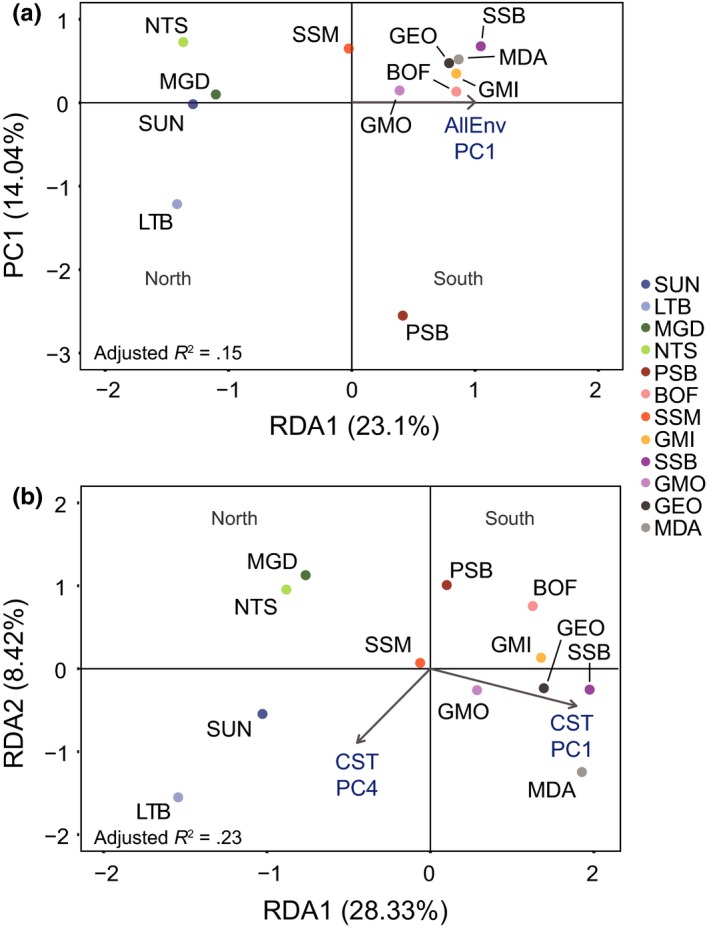
Redundancy analysis plots for loci detected as potentially under selection through environmental correlation with (a) AllEnv (90 environmental variables, *n* = 621 loci), (b) CST (36 environmental variables, *n* = 285 loci) in 12 populations of *Placopecten magellanicus*. Explanatory variables (arrows) were principal components axes from PCA on (a) AllEnv and (b) CST, retained as important following backwards stepwise variable selection

To examine the effects of climate versus geography on the genetic variation within the outlier SNP loci, we selected five PCs from AllEnvPCA and four from CSTPCA for use as explanatory variables in RDA, each explaining more than 5% of the total variance in the PCA. In AllEnvPCA, the five selected axes explained 89.78% of the total model variance, and in CSTPCA, the four selected axes explained 88.96% of the total variance. Backwards stepwise variable selection on the RDA for AllEnv retained only AllEnvPC1 as an important explanatory variable, whereas selection on the RDA for CST retained both CSTPC1 and CSTPC4 (Figure 7). Both models demonstrated significant relationships, despite low adjusted *R* ^2^ values (AllEnv, *R* ^2^_adj_ = .15, *p* = .001; CST, *R* ^2^_adj_ = .23, *p* = .001). Variance partitioning showed that climate explained a significant component of the model variation in both cases, explaining 32.36% of model variation in AllEnv (compared to 30.37% explained by geography and 37.28% explained as joint effects) and 41.34% of model variation in CST (compared to 21.27% explained by geography and 37.39% explained by joint effects).

**Figure 8:**
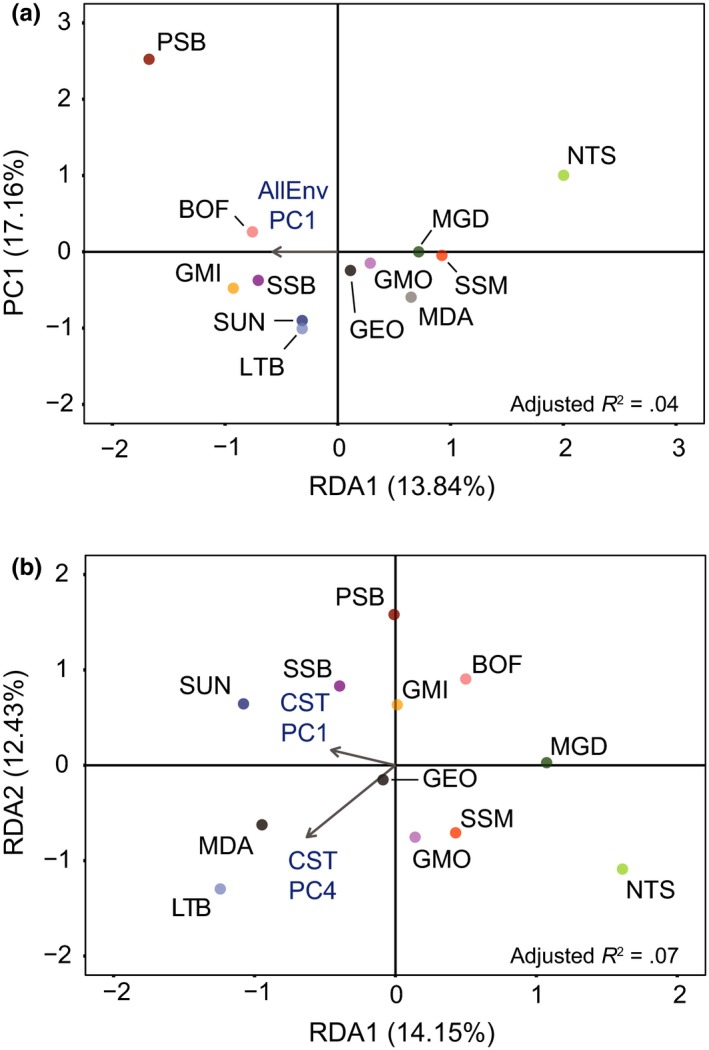
Partial redundancy analysis plots for loci detected as potentially under selection through environmental correlation with (a) AllEnv (90 environmental variables, *n* = 621 loci), (b) CST (36 environmental variables, *n* = 285 loci) in 12 populations of *Placopecten magellanicus*. Explanatory variables used were principal components axes from PCA on (a) AllEnv and (b) CST, retained following backwards stepwise variable selection. The genetic matrix was conditioned on the distance between populations to reduce the effects of geographic separation between populations

The RDAs for AllEnv and CST both separated north and south population groups. AllEnv retained only one environmental PC axis, and we therefore show only one RDA axis in the plot (Figure 7a); however, this axis clearly divides the north and south populations. In CST, RDA1 divided north and south but further division among sample sites can be seen along RDA2, including separation of populations from Newfoundland and the Gulf of St. Lawrence (Figure 7b). Partial RDA, following conditioning of the genetic matrix on the distance between populations, no longer separated north and south populations once the effect of population separation distance was removed (Figure 8, AllEnv, *R* ^2^_adj_ = .04, *p* = .06; CST, *R* ^2^_adj_ = .07, *p* = .03). We expected this result given the strong relationship between environmental parameters and latitude in this region and the large latitudinal but small longitudinal span of the samples.

To choose environmental parameters to include in the multiple linear models, we examined variable weightings on the PC axes selected during RDA and retained the five most highly weighted variables from each axis. For all variables included in each global model, we calculated cumulative AIC_c_ weights and model‐averaged parameter estimates (Table 7). Model selection using CSTEnv and all 10 selected environmental variables could not determine best fit models and provide accurate estimates for parameter weights and coefficients due to overfitting of the model. Upon further examination of the RDA results, CSTPC1 appeared more important in driving the north–south population split. We repeated our multiple linear regressions and model averaging using only the five most highly weighted variables from CSTEnvPC1. In all cases, model weights averaged over all possible iterations of the models containing a particular variable indicated surface average winter temperature as the most important variable. Surface minimum temperature (occurred in winter) and deep average winter temperature also ranked highly, suggesting that the coldest temperatures encountered by both juvenile and adult scallops may play an important structuring role for scallop populations. Parameter estimates for all three variables were positive; increased minimum temperatures in the model corresponded to larger values of the first PC (higher PC values match the south population cluster).

**Table 7:** Cumulative Akaike information criterion model weights (Σ ω_i_) and model‐averaged parameter estimates (full: variables assumed to be present in all models with a coefficient of 0 in some cases; subset: variables only present in models where the coefficient was not 0) in models predicting whether genetic variation in outlier loci among populations of *Placopecten magellanicus* is a function of environmental variation. (A) Outlier loci were detected through correlations with an environmental dataset of 90 variables (AllEnv, *n* = 621 loci). Environmental variables were selected following the results of principal components analysis and redundancy analysis. (B) Outlier loci were detected through correlations with an environmental dataset of 36 variables (CST, *n* = 285 loci). Environmental variables were selected following the results of principal components analysis and redundancy analysis. (C) Outlier loci were the same used in (A). Environmental variables were selected following the results from (A) and (B). (D) Outlier loci were the same used in (C). Environmental variables were selected following the results from (A) and (B)

	Parameter	Σ ω_i_	Model‐averaged parameter estimates	
			Full	Subset
(A)	SurfAvWinTemp	0.426	0.6767452	1.5889694
	SurfMinTemp	0.315	0.1673587	0.5337035
	SurfAvAutSal	0.272	0.2465678	0.9065121
	DepMinSiO_4_	0.256	0.2009489	0.7841225
	DepAvAutSal	0.152	0.02467925	0.16288929
(B)	SurfAvWinTemp	0.437	0.5586436	1.2785748
	DepAvWinTemp	0.344	0.5687936	1.6505098
	DepMaxSal	0.343	−0.4611794	−1.3452456
	DepMinTemp	0.275	0.3441115	1.2523863
	DepAvAutSal	0.109	−0.001612218	−0.014871888
(C)	SurfAvWinTemp	0.643	1.031135	1.604291
	DepAvWinTemp	0.282	0.04939479	0.17471122
	SurfAvAutTemp	0.142	0.1584858	1.0963318
	SurfAvSumTemp	0.141	−0.09856687	−0.69285742
	DepAvSprTemp	0.103	0.04809931	0.45494143
	DepAvAutTemp	0.095	0.04477454	0.46824153
	DepAvSumTemp	0.083	0.01956854	0.23171602
	SurfAvSprTemp	0.080	−0.01290984	−0.1627252
(D)	SurfAvWinTemp	0.587	0.6509583	1.10358
	DepAvWinTemp	0.302	0.1932762	0.6457666
	DepAvSprTemp	0.131	0.004951037	0.067160279
	SurfAvAutTemp	0.121	0.08023971	0.5923327
	SurfAvSumTemp	0.095	0.01497057	0.16543456
	DepAvAutTemp	0.092	−0.02916119	−0.30036982
	DepAvSumTemp	0.089	0.02933703	0.312122
	SurfAvSprTemp	0.072	0.07923188	0.63938784

**Figure 9:**
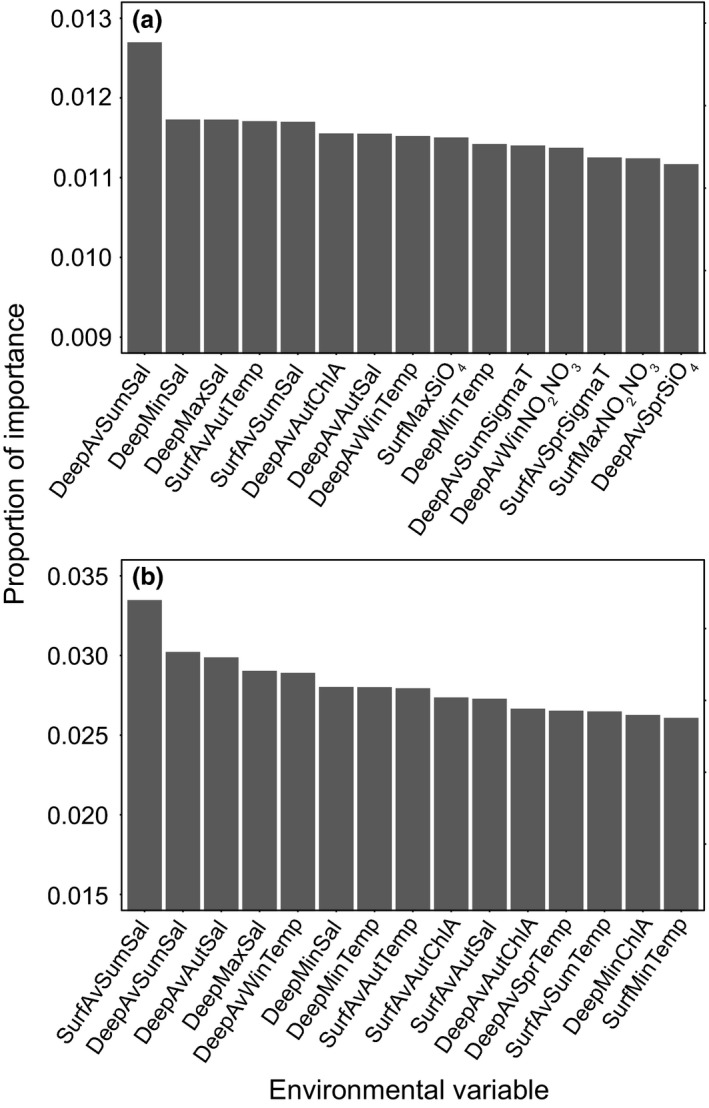
Proportion of importance (average per variable importance/importance sum of all variables) for the top 15 environmental variables determined using random forest and (a) AllEnv and AllEnvOutlier, and (b) CST and CSTOutlier

Using RF, we calculated the importance proportion for all environmental variables using both AllEnvOutlier and CST Outlier (Figure 9). Using AllEnvOutlier, deep average summer salinity, deep minimum salinity (occurred in spring), and deep maximum salinity (occurred in autumn) ranked as the most important environmental variables. Surface average autumn temperature, deep average winter temperature, and deep minimum temperature (occurred in winter) were also selected as important variables. CSTOutlier once again ranked salinity‐associated variables as most important; however, deep average winter temperature and deep minimum temperature ranked highly and the importance proportions for CSTOutlier exceeded those from AllEnvOutlier.

### 3.5 Gene ontology

Blast2GO functionally annotated very few outlier loci. CSTOutlier determined annotation matches for only four loci (1.4% of loci), with a BLAST hit but no GO annotation at one further locus. In AllEnvOutlier, only five loci (0.8% of total loci) matched, with a BLAST hit but no GO annotation in one further locus. The two lists of outliers shared three matches, with GO annotations split between molecular function (calcium ion and carbohydrate binding) and metabolic processes (regulation of transcription and steroid hormone‐mediated signaling). In CSTOutlier, GO annotation of the remaining locus identified a molecular function (oxidoreductase activity) and a metabolic process (oxidation–reduction process). In AllEnvOutlier, the GO annotations of the remaining two loci differed, one locus with molecular functions (oxidoreductase activity) and metabolic processes (oxidation–reduction process) and the other locus with several annotations (molecular function/catalytic activity, transferase activity, and folic acid binding, and metabolic processes/cellular metabolic processes) (Table 8).

**Table 8:** Blast2GO functional annotation of outlier SNP loci from 12 populations of *Placopecten magellanicus*. Outliers were detected through environmental correlations with 90 environmental variables (AllEnv, 621 loci) or a subset of 26 environmental variables (CST, 285 loci)

Environmental data	Locus name	GO name
AllEnv	16087_68	F: catalytic activity; P: metabolic process; F: transferase activity; F: folic acid binding; P: cellular metabolic processes
AllEnv	24384_24	F: oxidoreductase activity; P: metabolic process; P: oxidation–reduction process
AllEnv and CST	12228_13	P: regulation of transcription; P: steroid hormone‐mediated signaling pathway
AllEnv and CST	20561_41	F: carbohydrate binding
AllEnv and CST	25748_78	F: calcium ion binding
CST	15446_21	F: oxidoreductase activity, acting on paired donors, with incorporation or reduction of molecular oxygen, reduced pteridine as one donor, and incorporation of one atom of oxygen; P: oxidation–reduction process

## 4 DISCUSSION

The identification of environmental factors regulating marine population structure can both inform fisheries management through the identification of management units and help predict species' responses to environmental change. Here, we applied a landscape genomics approach using 7163 RAD‐seq‐derived SNPs (previously identified in Van Wyngaarden et al., 2017) and 90 environmental variables to identify oceanographic factors associated with a latitudinal genomic cline in sea scallops in eastern North America. Our results support the hypothesis that seasonal periods of extreme cold restrict gene flow and influence population structure in this species. This work builds on previous studies on population structure in *P. magellanicus* (Kenchington et al., 2006; Owen & Rawson, 2013), particularly the identification of a major genomic discontinuity separating the north and south of the species range (Van Wyngaarden et al., 2017). Our multivariate analysis using the outlier loci and environmental variables identified minimum and average winter temperatures as the most important variables describing genetic variation among populations of the scallop, indicating that overwinter survival may strongly influence structure of these populations. We also identified minimum salinity as a potential structuring force, although to a lesser extent and affecting fewer populations than temperature changes over the range of the species. Overall, the observed genomic and environmental correlations support the hypothesis of latitudinal structuring driven predominantly by ocean temperature.

### 4.1 Environmental variables driving adaptation

Our results highlight ocean temperature as a critical environmental factor contributing to population structuring of the sea scallop. The sea scallop's distribution spans almost 10° latitude encompassing an extremely large range of environmental conditions (approximately 5–10°C difference in temperatures year‐round), primarily caused by prevailing currents (Townsend et al., 2006). The Labrador Current, a cold Arctic current, flows south from the coasts of northern Canada and Greenland, splitting around Newfoundland and circulating through the Gulf of St. Lawrence (Townsend et al., 2006). In contrast, the warm Gulf Stream moves north from the Gulf of Mexico along the east coast of North America. These two currents meet and move roughly offshore around Nova Scotia, exposing scallop populations to large differences in water temperature (and other oceanographic variables) in different areas of their range (Townsend et al., 2006). Our environmental PCAs clearly detected the differences in environment associated with these currents. The first PC in our environmental PCAs illustrates the split between northern and southern populations and for both AllEnv and CST explains more than 40% of the variation in the environmental data. Using *F*_ST_‐based outlier detection methods, Van Wyngaarden et al. (2017) identified a strong genetic population separation related to these currents and the other oceanographic and environmental features in the region; populations in the north, generally exposed to colder temperatures, clustered separately from the southern populations, which are often exposed to warmer temperatures. This genetic split also clearly appears in the first PC of our genetic outlier PCAs, however the first PC in both of our analyses explains little variance, especially when compared with outliers used in Van Wyngaarden et al. (2017). This difference in explained variance among the genetic PCs likely results from the method used to detect outlier loci given some differences between the outlier lists detected here and in Van Wyngaarden et al. (2017) (Table S3). Van Wyngaarden et al. (2017) used BayeScan (Foll & Gaggiotti, 2008), which uses an *F*_ST_‐based method to detect outliers and generally selects the most divergent loci. In comparison, both BAYENV2 and LFMM use environmental correlations to detect outlier loci. Although these methods may also identify highly divergent loci as outliers, if a highly divergent locus (likely to be detected by BayeScan) does not correlate with the environmental variation captured in our environmental dataset, it would not be included in the final outlier list and would not contribute to the variance explained by the first PC in our genetic PCAs.

Van Wyngaarden et al. (2017) documented a genetic discontinuity between northern and southern scallop populations in all SNP loci, although the magnitude of differentiation was significantly higher at outlier loci. This pattern indicates that although neutral processes may play a role in population structuring, selection plays a dominant structuring role across the geographic range of the sea scallop. Considering this finding, we focused on environmental‐based outlier detection methods to identify putative causes of population structure in the sea scallop. Of the environmental variables we examined, temperature primarily drives the separation of northern and southern populations and the coldest temperatures (winter and minimum) differ most between these groups. This finding is consistent with other studies in the North Atlantic, where temperature variations (particularly with latitude) represent some of the strongest differences among regions (Townsend et al., 2006); temperature is likely the dominant selective force in this region and among scallop populations. Strong correlations between genetic variation and ocean temperatures have been observed in many North Atlantic fish species (e.g., Berg et al., 2015; Bourret, Dionne, Kent, Lien, & Bernatchez, 2013; Bradbury et al., 2010, 2014; Limborg et al., 2012) and other North Atlantic invertebrates, specifically in Benestan et al. (2016) where the population structure of American lobster (*Homarus americanus*) was also found to be driven by minimum annual water temperatures.

**Figure 10:**
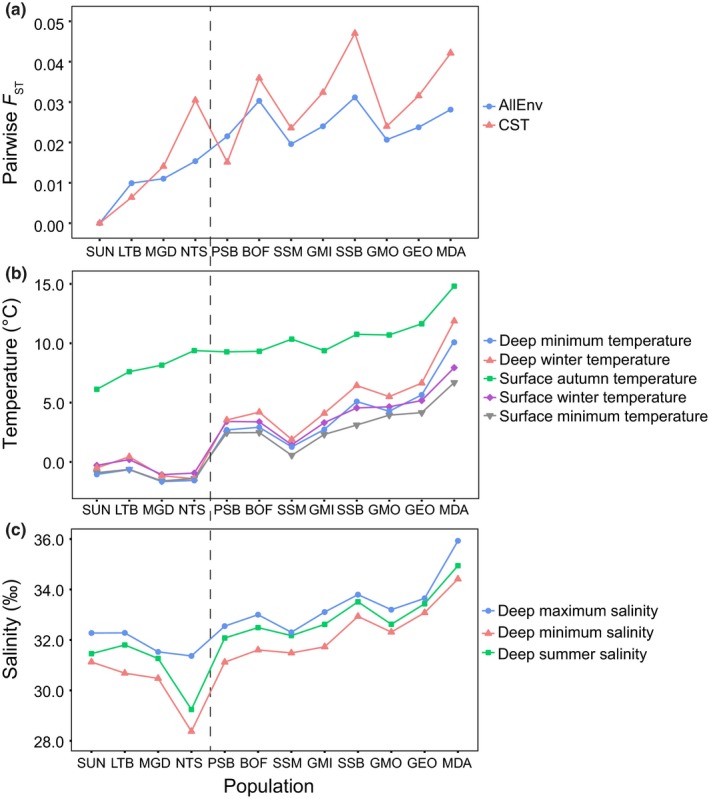
(a) Pairwise *F*_ST_ values calculated between all populations (ordered north to south) and the furthest north population (SUN). (b) Values of the temperature variables found to be important determinants of population structure using multiple linear regression and random‐forest analysis for all populations. (c) Values of the salinity variables found to be important determinants of population structure using random‐forest analysis for all populations. The dashed line represents the split between the northern (four populations) and southern (eight populations) population groups reported in Van Wyngaarden et al. (2017)

However, in many regions other environmental features often covary with temperature (e.g., salinity or ChlA) and in some analyses temperature may act as an unintentional proxy for the true selective force (a species may appear to adapt to temperature when in fact they are experiencing selection due to another variable such as ocean productivity). This may have particular relevance for the sea scallop, because our sampling locations and temperature gradient both span the same north–south axis. There is also some evidence of a slight north–south salinity gradient, with the lowest salinities in MGD and NTS and the highest salinity in MDA (Figure 6b,c). Although clear associations between genetic variation and temperature have been reported in several other species, including Pacific invertebrates (Pespeni & Palumbi, 2013), studies also demonstrate genomic adaptation to environmental gradients other than temperature, such as adaptation to salinity gradients in several Baltic Sea species (Berg et al., 2015; Limborg et al., 2012; Sjöqvist et al., 2015). In our analyses, in addition to cold temperatures RF analysis also identified salinity as an important environmental variable, likely driven by very low salinity values at NTS and MGD in the Gulf of St. Lawrence. Our RF analyses used allele frequencies across all populations; however, by handling covariation between environmental data RF may have been able to detect the smaller‐scale variation associated with salinity in the Gulf of St. Lawrence that may have been masked by the strong temperature associations influencing the multiple linear regression analysis. Overall, genetic variation reflects the geographic patterns present in the significant environmental variables (Figure 10). When plotted against pairwise *F*_ST_ (calculated using ARLEQUIN v.3.5 (Excoffier & Lischer, 2010)), the winter and minimum water temperatures clearly differ between Van Wyngaarden et al. (2017)'s northern and southern population groups. Additionally, NTS has a higher differentiation from SUN than other nearby populations, potentially reflecting a response to the lower minimum and summer salinities present at that location. Further sampling along a salinity gradients not confounded by a temperature gradient may help to disentangle the covarying effects.

### 4.2 Mechanisms of adaptation

The genomic associations with ocean temperature during periods of extreme cold (i.e., winter) suggest temperature‐associated mortality may significantly structure sea scallop populations. Sea scallops reproduce via broadcast spawning, generally in the autumn, although timing varies along their range. Given that scallops tend to spawn in the warmest water (Thompson, 1977), generally between August and October (Beninger, 1987; Langton, Robinson, & Schick, 1987; Naidu, 1970), and they likely settle before December (Naidu & Robert, 2006), a link between winter temperatures and larval mortality appears unlikely. Our analyses point to the overwinter survival of juvenile scallops as a potentially important structuring force limiting the effective dispersal of scallops between our northern and southern population groups, rather than selective mortality of planktonic larval scallops, and future experimental studies on larval and juvenile scallops may help to clarify this possibility. Some evidence suggests that temperatures experienced by adults can help ensure a healthy larval year class (Dickie, 1955; DuPaul, Kirkley, & Schmitzer, 1989; Kirkley & Dupaul, 1991; Langton et al., 1987; Macdonald & Thompson, 1985). Interestingly, our study identified surface temperature rather than temperature at depth as the most important driver of selection, contrary to expectations of juvenile scallop survival. One possible explanation is that deep temperature values are often estimated or provided as a range at collection sites, presumably reducing accuracy of those measurements relative to those for surface temperature. Our Blast2GO results identified possible genetic matches with several cellular processes, which may be temperature dependent, highlighting potential mechanisms of thermal adaptation in the sea scallop. Unfortunately, the lack of available genetic resources (i.e., reference genome) for the sea scallop impedes our ability to evaluate fully the functional importance of the loci identified here. Until improved genetic data resources for the sea scallop or related species are available, any conclusions drawn from annotation results are preliminary.

### 4.3 Alternative contributions to population structure

Despite the clear association observed with ocean temperature and population structure, selective processes may not be the sole driving mechanism of population structure in the sea scallop. Neutral oceanographic barriers to connectivity have been documented in other marine bivalves in the Northwest Atlantic, including *Mytilus sp*. in the Gulf of Maine (Yund et al.*,* 2015). As described in a review by Bierne, Welch, Loire, Bonhomme, and David (2011), in many cases local adaptation alone may not explain the genetic structure detected among populations or the geographic location of the strong break between population clusters. Tension zones (caused by endogenous barriers to gene flow) may have arisen independent of selection caused by environmental variation along the range of the species, potentially influencing the separation of population groups between the north and south of the species range. These tension zones may associate with environmental clines, and a combination of both endogenous and exogenous barriers (tension zones and selection) could contribute to the detected structure. This scenario could also reinforce local adaptation associated with environmental adaptation (Sexton, Hangartner, & Hoffmann, 2014; Shafer & Wolf, 2013), furthering differentiation between regions. The detection of a genetic discontinuity between northern and southern populations of the sea scallop in both outlier and neutral loci (Van Wyngaarden et al., 2017) suggests that a combination of neutral and selective forces leads to the population structure detected, although the magnitude of the genetic break in outlier loci is larger than that of neutral loci indicating that selective forces play a larger structuring role than neutral forces in the sea scallop.

The sea scallop range encompasses an area of complex oceanography and several processes could contribute to the neutral genetic separation of populations including potential current‐related fronts that may prevent larval movement between regions, upwelling and water movement related to the continental shelf, and storm mixing along the coast (Townsend et al., 2006). These factors may inhibit the successful movement, settlement, and growth of larvae and can be difficult to accurately incorporate into models of connectivity that try to calculate larval dispersal. Additionally, larval behavior significantly impacts dispersal in many cases (Shanks, 2009) and can be difficult to accurately model and evaluate. Using postsettlement genetic structure to determine what processes influence connectivity among sea scallop populations, our methods inherently account for the effects of larval behavior and complexity when drawing conclusions. Although we believe our results to be robust to complications of neutral population structure and geographic distance, additional sampling (especially from populations at the same latitude) will help to more thoroughly separate the joint effects of climate and geography on scallop population structure as it may allow sampling of populations with a similar climatic profile at varying separation distances.

### 4.4 Challenges and limitations

Many reviews on environmental association studies recommend removing the effects of neutral population structure to fully assess the effect of selection on population structure in natural systems (e.g., Rellstab, Gugerli, Eckert, Hancock, & Holderegger, 2015) and accounting for geographic distance and isolation by distance when examining potential isolation by ecology (e.g., Shafer & Wolf, 2013); however, this is a particular challenge in our system. Because a single north–south population split characterizes our sample sites rather than a classic isolation‐by‐distance pattern (Van Wyngaarden et al., 2017), geographic distance among populations may not influence our results the way it would in a system characterized by a classic stepping‐stone pattern. Our samples also align along the north–south axis of the population range providing few opportunities to examine the effects of distance between samples without also removing the effects of latitude. To minimize the potential bias of neutral population structure on our results, we focused our analysis solely on outlier loci potentially under selection in the genome, likely making our analyses less prone to the confounding effects of neutral population structure. We also compared the results of RDA and pRDA, which controls for geographic distance among populations. Even when controlling for geographic distance, our results nonetheless indicate climate as a significant population structuring force, although the patterns of population clustering change slightly.

Another source of bias in population genomics studies is the effect of age‐related structuring in population samples, which has been documented in scallops previously (Owen & Rawson, 2013), potentially due to recruitment events or yearly environmental fluctuations. Although our samples were collected over a relatively short time period (2011–2013), we made attempts to cover multiple age classes to avoid this issue.

Our analyses pinpointed potential environmental influences on sea scallop population structure; however, annotating the outlier SNPs of interest remains challenging. Although RAD‐seq generates vast quantities of SNPs in organisms without reference genomes (Benestan et al., 2015; Catchen et al., 2013; Hohenlohe et al., 2012; Reitzel, Herrera, Layden, Martindale, & Shank, 2013), the lack of more detailed genetic resources makes inference on the causal mechanisms contributing to local adaptations in sea scallops difficult, as noted by our lack of GO matches. Fortunately, with continued development of resources for *P. magellanicus* and related species, future studies will likely identify and study the features most important in characterizing sea scallop population structure.

## 5 CONCLUSIONS

Our results show that ocean climate plays a role in structuring populations of sea scallops, particularly the influence of the coldest temperatures experienced. The association with coldest temperatures points to the overwinter survival of juvenile scallops as a structuring force rather than survival of larval scallops, contrary to what might be expected for broadcast spawning marine species. This work and similar landscape (or seascape) genetic studies highlight the possibility that local adaptation and the differential survival of dispersers (rather than solely limited dispersal) may have greater impact on the population structure of marine species than previously hypothesized. Our results can be useful in the effective management of *P. magellanicus* by helping managers in both Canada and the United States accurately determine geographic sources of larvae for exploited populations and predict the potential reactions of this species to a changing ocean climate, particularly with changes to the location and strength of dominant currents. Our results also provide an important starting point for future studies. If temperature drives variation in the reproductive rates of scallops, then increasing water temperatures associated with global warming may alter scallop reproductive cycles and subsequent recruitment (Robinson, Martin, Chandler, & Parsons, 2007). Genetic and genomic studies to examine further effects of selection on population structure in scallops, in tandem with experimental studies to identify adaptations among scallop populations, may be critical for predicting how the species will react to future climate change and harvesting pressures. Additionally, access to further genetic resources will continue to improve identification of the genes and pathways involved in adaptation and population structuring among sea scallop populations.

## DATA ACCESSIBILITY

All raw sequences are available at NCBI SRA Bioproject number PRJNA340326, Biosample numbers SAMN05712457–SAMN05712468. The full outlier lists are available in Tables S1 and S2. The sample site distance matrices, final environmental metrics, the 7,216 SNPs selected before HWE filtering (VCF format), sequence sample metadata, and annotated R scripts of the main statistical analyses are available on Dryad at http://datadryad.org/resource/doi:10.5061/dryad.c15v5.

## CONFLICT OF INTEREST

None declared.

## AUTHOR CONTRIBUTIONS

IB, PS, and CDB planned and coordinated scallop sample collection. LH completed the sample preparation and RAD‐seq library preparation. NRE processed the raw RAD‐seq data and prepared the final genotype tables. MVW, IB, PS, and CDB planned the analyses. MVW completed the analysis and wrote the manuscript. IB, PS, and CDB read, edited, and approved the manuscript. LZ completed the random‐forest analysis with guidance and advice from RGB. All authors submitted comments, edits, and suggestions during the final preparation of the manuscript.
