# Supplementary material for: Oceanographic variation influences spatial genomic structure in the sea scallop, Placopecten magellanicus
Source: Ecol Evol. 2018 Feb 11;8(5):2824–41. doi: 10.1002/ece3.3846 (PMC5838053; doi:10.1002/ece3.3846)
Supplement: Supplementary file 1 [file ECE3-8-2824-s001.doc]

**Supporting Information**

for

**Oceanographic variation influences spatial genomic structure in the sea scallop, *Placopecten magellanicus***

| Table S1. Comparison of outlier SNP loci from 12 populations of *P. magellanicus* determined using BayEnv2 and a dataset of 90 environmental variables (AllEnv, 128 loci) and a subset of 36 environmental variables (CST, 72 loci). Shared loci are italicized. | |
| --- | --- |
| **AllEnv** | **CST** |
| 319_43 | 479_44 |
| 479_15 | 545_13 |
| *1089_23* | *1089_23* |
| *1299_57* | *1299_57* |
| *1320_61* | *1320_61* |
| *2272_32* | *2272_32* |
| 2966_52 | 2305_45 |
| 3061_77 | 2694_52 |
| 3069_13 | *3240_12* |
| 3199_76 | 3240_55 |
| *3240_12* | *3240_56* |
| *3240_56* | *3929_9* |
| 3299_78 | 4484_19 |
| 3350_55 | *4668_81* |
| 3420_33 | 4769_32 |
| 3498_66 | 5062_61 |
| 3619_79 | 5353_70 |
| 3834_87 | *5439_27* |
| *3929_9* | 6968_50 |
| 4063_7 | 7115_35 |
| 4383_42 | 7325_7 |
| *4668_81* | 7326_20 |
| 4847_58 | 8388_15 |
| 4975_23 | 8664_7 |
| 5045_48 | *9554_49* |
| 5084_54 | 9881_90 |
| 5084_64 | *10070_67* |
| 5161_82 | *10832_84* |
| 5247_15 | *11531_7* |
| 5420_31 | 12021_80 |
| *5439_27* | *12155_52* |
| 6805_6 | *12314_60* |
| 7120_90 | *12767_68* |
| 7203_45 | *13384_35* |
| 7343_38 | 13561_17 |
| 7361_21 | 13561_70 |
| 7882_64 | 13761_22 |
| 7997_36 | 13805_16 |
| 8325_27 | *13824_83* |
| 8655_19 | *13891_65* |
| 8782_41 | 14003_84 |
| 8922_62 | *14197_90* |
| 9203_74 | *14394_27* |
| 9431_86 | 15121_78 |
| *9554_49* | 15446_21 |
| 9653_60 | *15645_89* |
| 9976_51 | *15821_29* |
| *10070_67* | *16110_74* |
| 10159_36 | 16229_44 |
| 10366_19 | 17320_31 |
| 10498_47 | 17856_41 |
| 10529_34 | *18391_26* |
| 10832_74 | *18439_32* |
| *10832_84* | 18645_34 |
| 10917_59 | *18669_56* |
| 10987_36 | 19330_41 |
| 11265_39 | 19880_7 |
| *11531_7* | *20263_79* |
| 11748_62 | *20298_27* |
| 12014_82 | *20298_73* |
| *12155_52* | 20311_14 |
| *12314_60* | 20633_88 |
| *12767_68* | 20810_86 |
| 13260_51 | *21297_65* |
| *13384_35* | *22123_81* |
| 13438_71 | *22237_35* |
| 13744_54 | 25114_12 |
| *13824_83* | *25405_78* |
| *13891_65* | *25627_51* |
| 14076_10 | 25646_16 |
| 14197_86 | *25748_78* |
| *14197_90* | *26519_27* |
| *14394_27* |  |
| 14421_56 |  |
| 14557_41 |  |
| 14571_64 |  |
| 15171_47 |  |
| 15245_72 |  |
| 15459_62 |  |
| *15645_89* |  |
| 15660_9 |  |
| 15722_73 |  |
| *15821_29* |  |
| 15876_15 |  |
| *16110_74* |  |
| 16161_6 |  |
| 16206_40 |  |
| 16309_22 |  |
| 16393_64 |  |
| 16517_82 |  |
| 16943_74 |  |
| 17480_65 |  |
| 17720_35 |  |
| 17853_46 |  |
| 18135_35 |  |
| 18238_55 |  |
| *18391_26* |  |
| *18439_32* |  |
| *18669_56* |  |
| 19353_39 |  |
| 19487_24 |  |
| 19960_23 |  |
| 20047_88 |  |
| *20263_79* |  |
| *20298_27* |  |
| *20298_73* |  |
| 20548_19 |  |
| 20596_34 |  |
| 20910_86 |  |
| *21297_65* |  |
| 21600_62 |  |
| 21679_27 |  |
| 21717_37 |  |
| *22123_81* |  |
| *22237_35* |  |
| 23535_28 |  |
| 23969_73 |  |
| 24257_63 |  |
| 24939_35 |  |
| 25067_48 |  |
| *25405_78* |  |
| 25405_86 |  |
| *25627_51* |  |
| 25630_84 |  |
| *25748_78* |  |
| *26519_27* |  |
| 26726_84 |  |
| 27045_28 |  |

| Table S2**.** Comparison of outlier SNP loci from 12 populations of *P. magellanicus* determined using latent factor mixed models and a dataset of 90 environmental variables (AllEnv, 511 loci) and a subset of 36 environmental variables (CST, 218 loci). Shared loci are italicized. | |
| --- | --- |
| **AllEnv** | **CST** |
| 85_6 | *370_51* |
| 367_35 | *370_60* |
| 367_88 | *618_44* |
| *370_51* | *620_25* |
| *370_60* | *1115_68* |
| 418_86 | *1122_21* |
| 450_37 | *1463_75* |
| *618_44* | *1463_88* |
| *620_25* | *1471_75* |
| 620_53 | *1484_21* |
| 874_57 | *1714_38* |
| 1115_23 | *1867_71* |
| *1115_68* | *2450_62* |
| *1122_21* | *2670_59* |
| 1129_54 | *2809_37* |
| 1143_46 | *3216_12* |
| 1383_82 | *3303_73* |
| 1459_43 | *3895_8* |
| *1463_75* | *3895_88* |
| *1463_88* | *4066_45* |
| *1471_75* | *4186_46* |
| *1484_21* | *4546_80* |
| 1566_34 | *4613_35* |
| 1683_20 | *4665_62* |
| 1689_82 | *4668_42* |
| *1714_38* | *4668_81* |
| 1742_90 | *4844_25* |
| 1743_28 | *4956_31* |
| 1847_71 | *4970_90* |
| 1856_80 | *4975_9* |
| 1856_82 | *4975_68* |
| *1867_71* | *5170_73* |
| 1974_44 | *5204_27* |
| 2334_90 | *5354_34* |
| 2395_23 | *5419_44* |
| *2450_62* | *5522_80* |
| *2670_59* | *5842_16* |
| *2809_37* | *5978_53* |
| 3000_6 | *6026_30* |
| 3028_9 | *6026_53* |
| 3124_7 | *6190_47* |
| 3124_74 | *6491_67* |
| 3124_8 | *6579_25* |
| *3216_12* | *6591_67* |
| 3286_90 | *6645_6* |
| *3303_73* | *6688_49* |
| 3381_74 | *6976_68* |
| 3420_33 | *7042_35* |
| *3895_8* | *7376_36* |
| *3895_88* | *7408_7* |
| 3922_74 | *7648_39* |
| 3922_84 | *7724_27* |
| 3936_13 | *7846_50* |
| 3945_18 | *7941_58* |
| 3975_33 | *7999_74* |
| 4004_43 | *8380_74* |
| *4066_45* | *8520_42* |
| *4186_46* | *8575_6* |
| 4320_27 | *8575_39* |
| *4546_80* | *8794_9* |
| *4613_35* | *8797_34* |
| *4665_62* | *8836_15* |
| 4667_77 | *8847_48* |
| *4668_42* | *8849_28* |
| 4668_66 | *9076_28* |
| *4668_81* | *9076_54* |
| 4729_44 | *9098_83* |
| 4832_89 | *9169_48* |
| *4844_25* | *9196_22* |
| *4956_31* | *9196_23* |
| *4970_90* | *9196_87* |
| 4975_23 | *9275_68* |
| *4975_68* | *9275_76* |
| *4975_9* | *9275_79* |
| 5070_52 | *9275_81* |
| *5170_73* | *9508_72* |
| *5204_27* | *10464_33* |
| 5298_76 | *10464_52* |
| *5354_34* | *10681_10* |
| 5363_56 | *10987_75* |
| 5394_86 | *11131_89* |
| *5419_44* | *11217_9* |
| 5443_24 | *11315_68* |
| 5443_32 | *11634_72* |
| 5443_6 | *11909_85* |
| 5443_67 | *11989_13* |
| 5515_42 | *12014_82* |
| *5522_80* | *12228_13* |
| 5807_51 | *12236_9* |
| *5842_16* | *12314_60* |
| *5978_53* | *12363_39* |
| *6026_30* | *12580_62* |
| *6026_53* | *12639_88* |
| 6186_46 | *12651_47* |
| *6190_47* | *12680_26* |
| 6191_10 | *12880_57* |
| 6203_48 | *12884_30* |
| 6228_45 | *13012_81* |
| 6228_84 | *13075_87* |
| 6236_60 | *14197_88* |
| 6288_14 | *14197_90* |
| 6346_68 | *14316_32* |
| *6491_67* | *14326_62* |
| *6579_25* | *14415_20* |
| *6591_67* | *14532_68* |
| 6604_68 | *14564_90* |
| *6645_6* | *15508_47* |
| *6688_49* | *15638_70* |
| 6759_11 | *15759_32* |
| 6810_7 | *15759_54* |
| 6810_79 | *15903_58* |
| 6887_79 | *16388_50* |
| 6888_15 | *16442_35* |
| 6888_38 | *16442_83* |
| 6945_90 | *16442_86* |
| *6976_68* | *16470_49* |
| *7042_35* | *16691_70* |
| 7114_22 | *16771_68* |
| 7318_31 | *16961_32* |
| *7376_36* | *17189_71* |
| *7408_7* | *17191_81* |
| 7423_59 | *17259_54* |
| 7423_71 | *17259_55* |
| 7540_65 | *17369_30* |
| 7626_32 | *17377_63* |
| 7637_73 | *17406_14* |
| *7648_39* | *17691_29* |
| 7700_76 | *17793_42* |
| *7724_27* | *17865_54* |
| 7724_45 | *17939_43* |
| 7737_36 | *18011_51* |
| *7846_50* | *18013_68* |
| 7857_24 | *18084_42* |
| 7857_32 | *18084_67* |
| 7857_74 | *18175_82* |
| 7857_8 | *18180_41* |
| *7941_58* | *18180_85* |
| *7999_74* | *18180_90* |
| *8380_74* | *18272_54* |
| *8520_42* | *18286_51* |
| 8540_90 | *18411_69* |
| 8563_90 | *18473_63* |
| *8575_39* | *18555_47* |
| *8575_6* | *18669_56* |
| 8635_12 | *18692_21* |
| 8735_68 | *18692_22* |
| 8741_86 | *18800_64* |
| 8794_56 | *18832_29* |
| *8794_9* | *18968_21* |
| *8797_34* | *19146_65* |
| 8797_89 | *19197_77* |
| *8836_15* | *19256_31* |
| *8847_48* | *19284_7* |
| *8849_28* | *19284_8* |
| *9076_28* | *19478_77* |
| 9076_31 | *19738_6* |
| *9076_54* | *19738_49* |
| *9098_83* | *19752_32* |
| 9163_31 | *19844_50* |
| *9169_48* | *20070_71* |
| *9196_22* | *20140_47* |
| *9196_23* | *20314_14* |
| *9196_87* | *20314_46* |
| 9211_22 | *20556_7* |
| *9275_68* | *20556_14* |
| *9275_76* | *20556_31* |
| *9275_79* | *20559_46* |
| *9275_81* | *20561_41* |
| 9281_51 | *20721_17* |
| 9387_50 | *20868_9* |
| 9387_7 | *20868_67* |
| 9431_43 | *20885_25* |
| 9431_86 | *20933_88* |
| 9471_77 | *21167_6* |
| *9508_72* | *21362_67* |
| 9996_36 | *21381_83* |
| 10141_55 | *21413_20* |
| 10156_85 | *21413_21* |
| 10361_56 | *21413_48* |
| *10464_33* | *21503_20* |
| *10464_52* | *21696_25* |
| 10485_37 | *22000_20* |
| 10498_47 | *22000_86* |
| 10527_56 | *22011_10* |
| 10587_84 | *22011_82* |
| *10681_10* | *23849_13* |
| 10810_32 | *24019_69* |
| 10832_30 | *24336_71* |
| 10832_46 | *24406_47* |
| 10832_74 | *24509_20* |
| 10832_84 | *24636_63* |
| 10873_33 | *24673_67* |
| 10987_74 | *24892_12* |
| *10987_75* | *24908_10* |
| 10987_8 | *25107_50* |
| 10993_65 | *25246_11* |
| 11126_44 | *25380_52* |
| *11131_89* | *25380_84* |
| *11217_9* | *25405_6* |
| *11315_68* | *25405_9* |
| 11407_80 | *25433_29* |
| 11431_85 | *25433_34* |
| *11634_72* | *25433_45* |
| 11696_20 | *25433_82* |
| 11696_43 | *25627_51* |
| 11784_88 | *25993_87* |
| 11810_49 | *26222_72* |
| *11909_85* | *26293_14* |
| 11966_39 | *26340_40* |
| *11989_13* | *26431_8* |
| *12014_82* | *26431_77* |
| 12031_71 | *26625_75* |
| 12041_65 | *26721_7* |
| *12228_13* | *26721_89* |
| *12236_9* | *26781_56* |
| *12314_60* | *26880_82* |
| *12363_39* | *26952_89* |
| 12370_76 | *27011_89* |
| 12370_81 |  |
| 12377_67 |  |
| 12419_58 |  |
| *12580_62* |  |
| *12639_88* |  |
| *12651_47* |  |
| 12651_75 |  |
| 12674_8 |  |
| *12680_26* |  |
| *12880_57* |  |
| *12884_30* |  |
| 12936_75 |  |
| 12969_14 |  |
| 12969_15 |  |
| 12969_59 |  |
| *13012_81* |  |
| 13020_90 |  |
| *13075_87* |  |
| 13109_21 |  |
| 13197_42 |  |
| 13270_21 |  |
| 13286_17 |  |
| 13303_49 |  |
| 13303_64 |  |
| 13303_79 |  |
| 13374_27 |  |
| 13438_71 |  |
| 13586_43 |  |
| 13713_14 |  |
| 13727_6 |  |
| 13727_69 |  |
| 13758_31 |  |
| 13966_68 |  |
| 13983_54 |  |
| 14018_57 |  |
| 14050_63 |  |
| 14117_19 |  |
| 14117_7 |  |
| 14139_22 |  |
| 14139_68 |  |
| 14185_21 |  |
| *14197_88* |  |
| *14197_90* |  |
| 14287_61 |  |
| 14310_37 |  |
| 14316_11 |  |
| *14316_32* |  |
| *14326_62* |  |
| *14415_20* |  |
| *14532_68* |  |
| *14564_90* |  |
| 14674_22 |  |
| 14967_65 |  |
| 15022_55 |  |
| 15090_47 |  |
| 15203_19 |  |
| 15205_46 |  |
| 15205_67 |  |
| 15261_65 |  |
| 15301_34 |  |
| *15508_47* |  |
| 15638_54 |  |
| *15638_70* |  |
| 15687_33 |  |
| *15759_32* |  |
| *15759_54* |  |
| 15900_50 |  |
| 15903_35 |  |
| *15903_58* |  |
| 15903_76 |  |
| 15903_78 |  |
| 16087_68 |  |
| 16161_58 |  |
| 16161_6 |  |
| 16161_63 |  |
| 16161_64 |  |
| 16232_32 |  |
| 16232_64 |  |
| 16300_20 |  |
| 16339_61 |  |
| 16356_41 |  |
| *16388_50* |  |
| 16396_57 |  |
| *16442_35* |  |
| *16442_83* |  |
| *16442_86* |  |
| 16466_34 |  |
| *16470_49* |  |
| *16691_70* |  |
| *16771_68* |  |
| 16809_40 |  |
| 16837_37 |  |
| 16869_84 |  |
| 16891_32 |  |
| *16961_32* |  |
| *17189_71* |  |
| *17191_81* |  |
| *17259_54* |  |
| *17259_55* |  |
| *17369_30* |  |
| *17377_63* |  |
| 17382_16 |  |
| *17406_14* |  |
| 17466_32 |  |
| 17581_60 |  |
| 17581_65 |  |
| 17632_52 |  |
| *17691_29* |  |
| 17731_31 |  |
| *17793_42* |  |
| 17819_48 |  |
| *17865_54* |  |
| 17929_79 |  |
| 17929_9 |  |
| *17939_43* |  |
| 17998_24 |  |
| *18011_51* |  |
| *18013_68* |  |
| *18084_42* |  |
| *18084_67* |  |
| *18175_82* |  |
| 18178_75 |  |
| *18180_41* |  |
| *18180_85* |  |
| *18180_90* |  |
| 18238_55 |  |
| 18238_56 |  |
| *18272_54* |  |
| *18286_51* |  |
| 18296_75 |  |
| *18411_69* |  |
| 18444_28 |  |
| *18473_63* |  |
| 18481_72 |  |
| *18555_47* |  |
| 18642_16 |  |
| *18669_56* |  |
| *18692_21* |  |
| *18692_22* |  |
| 18701_58 |  |
| 18701_60 |  |
| 18701_83 |  |
| *18800_64* |  |
| *18832_29* |  |
| 18865_67 |  |
| *18968_21* |  |
| 18972_74 |  |
| *19146_65* |  |
| *19197_77* |  |
| 19218_80 |  |
| 19252_8 |  |
| *19256_31* |  |
| 19284_24 |  |
| 19284_32 |  |
| *19284_7* |  |
| *19284_8* |  |
| 19302_50 |  |
| 19330_39 |  |
| *19478_77* |  |
| 19654_11 |  |
| *19738_49* |  |
| *19738_6* |  |
| *19752_32* |  |
| 19819_35 |  |
| *19844_50* |  |
| 19898_23 |  |
| 19898_6 |  |
| 19898_61 |  |
| 20015_23 |  |
| 20047_71 |  |
| 20047_79 |  |
| 20047_87 |  |
| 20062_71 |  |
| *20070_71* |  |
| 20106_11 |  |
| 20117_82 |  |
| *20140_47* |  |
| *20314_14* |  |
| *20314_46* |  |
| 20527_29 |  |
| *20556_14* |  |
| *20556_31* |  |
| 20556_59 |  |
| *20556_7* |  |
| *20559_46* |  |
| *20561_41* |  |
| 20641_46 |  |
| *20721_17* |  |
| 20868_56 |  |
| *20868_67* |  |
| *20868_9* |  |
| *20885_25* |  |
| 20929_40 |  |
| *20933_88* |  |
| 20942_64 |  |
| 20955_8 |  |
| 21028_28 |  |
| 21125_75 |  |
| 21138_65 |  |
| *21167_6* |  |
| *21362_67* |  |
| *21381_83* |  |
| *21413_20* |  |
| *21413_21* |  |
| *21413_48* |  |
| 21418_49 |  |
| 21468_29 |  |
| 21468_89 |  |
| *21503_20* |  |
| 21600_62 |  |
| *21696_25* |  |
| 21746_21 |  |
| 21762_13 |  |
| 21853_20 |  |
| *22000_20* |  |
| *22000_86* |  |
| *22011_10* |  |
| *22011_82* |  |
| 22100_34 |  |
| 22122_62 |  |
| 23183_26 |  |
| 23296_73 |  |
| 23306_43 |  |
| 23346_81 |  |
| 23361_35 |  |
| *23849_13* |  |
| *24019_69* |  |
| 24230_82 |  |
| *24336_71* |  |
| 24373_14 |  |
| 24380_74 |  |
| 24384_24 |  |
| *24406_47* |  |
| *24509_20* |  |
| 24587_47 |  |
| *24636_63* |  |
| *24673_67* |  |
| 24804_65 |  |
| 24882_77 |  |
| *24892_12* |  |
| 24892_33 |  |
| *24908_10* |  |
| 24949_72 |  |
| 24954_38 |  |
| 25064_55 |  |
| *25107_50* |  |
| *25246_11* |  |
| 25310_43 |  |
| 25310_48 |  |
| 25366_26 |  |
| *25380_52* |  |
| 25380_58 |  |
| *25380_84* |  |
| *25405_6* |  |
| 25405_86 |  |
| *25405_9* |  |
| *25433_29* |  |
| *25433_34* |  |
| *25433_45* |  |
| *25433_82* |  |
| 25464_47 |  |
| 25562_55 |  |
| 25580_62 |  |
| 25599_88 |  |
| *25627_51* |  |
| 25630_84 |  |
| 25652_55 |  |
| *25993_87* |  |
| 26091_42 |  |
| 26091_9 |  |
| 26141_15 |  |
| 26141_84 |  |
| *26222_72* |  |
| *26293_14* |  |
| 26293_68 |  |
| 26312_54 |  |
| 26337_72 |  |
| *26340_40* |  |
| 26371_65 |  |
| 26391_35 |  |
| 26391_71 |  |
| *26431_77* |  |
| *26431_8* |  |
| 26550_72 |  |
| 26570_14 |  |
| *26625_75* |  |
| *26721_7* |  |
| *26721_89* |  |
| *26781_56* |  |
| *26880_82* |  |
| 26937_69 |  |
| *26952_89* |  |
| *27011_89* |  |

| Table S3**.** Comparison of outlier SNP loci from 12 populations of P. magellanicus determined using latent factor mixed models and BAYENV2 and a dataset of 90 environmental variables (AllEnv, 621 loci) and a subset of 36 environmental variables (CST, 285 loci) and 112 outlier SNP loci detected using *F*_ST_-based methods as reported in Van Wyngaarden, *et al*. (2017). Loci shared between the *F*_ST_-based outliers and either AllEnv or CST are italicized, loci shared between all three lists are in bold. | | |
| --- | --- | --- |
| **AllEnv** | **CST** | ***F*_ST_ outliers** |
| 85_6 | 370_51 | 11_6 |
| *319_43* | 370_60 | *319_43* |
| 367_35 | 479_44 | 979_75 |
| 367_88 | 545_13 | **1089_23** |
| 370_51 | 618_44 | **1299_57** |
| 370_60 | 620_25 | *3299_78* |
| 418_86 | **1089_23** | *3350_55* |
| 450_37 | 1115_68 | 3350_66 |
| 479_15 | 1122_21 | 3497_39 |
| 618_44 | **1299_57** | *3498_66* |
| 620_25 | 1320_61 | *3619_79* |
| 620_53 | 1463_75 | *3834_87* |
| 874_57 | 1463_88 | **3929_9** |
| **1089_23** | 1471_75 | 3969_43 |
| 1115_23 | 1484_21 | *4484_19* |
| 1115_68 | 1714_38 | **4668_81** |
| 1122_21 | 1867_71 | *4847_58* |
| 1129_54 | 2272_32 | **4975_9** |
| 1143_46 | 2305_45 | *4975_23* |
| **1299_57** | 2450_62 | **4975_68** |
| 1320_61 | 2670_59 | 5246_66 |
| 1383_82 | 2694_52 | 5252_37 |
| 1459_43 | 2809_37 | **5439_27** |
| 1463_75 | 3216_12 | *5515_42* |
| 1463_88 | 3240_12 | 5750_64 |
| 1471_75 | 3240_55 | 6948_62 |
| 1484_21 | 3240_56 | *7203_45* |
| 1566_34 | 3303_73 | *7326_20* |
| 1683_20 | 3895_8 | 7396_78 |
| 1689_82 | 3895_88 | 7524_34 |
| 1714_38 | **3929_9** | 7740_53 |
| 1742_90 | 4066_45 | 8699_63 |
| 1743_28 | 4186_46 | *8782_41* |
| 1847_71 | *4484_19* | 9206_48 |
| 1856_80 | 4546_80 | **9554_49** |
| 1856_82 | 4613_35 | 9580_74 |
| 1867_71 | 4665_62 | *9976_51* |
| 1974_44 | 4668_42 | 9976_52 |
| 2272_32 | **4668_81** | 9978_28 |
| 2334_90 | 4769_32 | 10349_43 |
| 2395_23 | 4844_25 | *10366_19* |
| 2450_62 | 4956_31 | *10498_47* |
| 2670_59 | 4970_90 | *10832_30* |
| 2809_37 | **4975_68** | *10832_46* |
| 2966_52 | **4975_9** | *10832_74* |
| 3000_6 | 5062_61 | **10832_84** |
| 3028_9 | 5170_73 | *10987_36* |
| 3061_77 | 5204_27 | 10987_48 |
| 3069_13 | 5353_70 | 11162_70 |
| 3124_7 | 5354_34 | **11531_7** |
| 3124_74 | 5419_44 | 12073_7 |
| 3124_8 | **5439_27** | 12308_45 |
| 3199_76 | 5522_80 | **12767_68** |
| 3216_12 | 5842_16 | **13384_35** |
| 3240_12 | 5978_53 | **13891_65** |
| 3240_56 | 6026_30 | **14394_27** |
| 3286_90 | 6026_53 | *14571_64* |
| *3299_78* | 6190_47 | 14750_60 |
| 3303_73 | 6491_67 | 15099_34 |
| *3350_55* | 6579_25 | *15446_21* |
| 3381_74 | 6591_67 | **15645_89** |
| 3420_33 | 6645_6 | *15660_9* |
| *3498_66* | 6688_49 | **15821_29** |
| *3619_79* | 6968_50 | **16110_74** |
| *3834_87* | 6976_68 | 16228_63 |
| 3895_8 | 7042_35 | *16229_44* |
| 3895_88 | *7115_35* | *16309_22* |
| 3922_74 | 7325_7 | 16478_29 |
| 3922_84 | *7326_20* | 16894_73 |
| **3929_9** | 7376_36 | 17085_50 |
| 3936_13 | 7408_7 | 17567_80 |
| 3945_18 | 7648_39 | *18135_35* |
| 3975_33 | 7724_27 | **18391_26** |
| 4004_43 | 7846_50 | **18669_56** |
| 4063_7 | 7941_58 | 19165_26 |
| 4066_45 | 7999_74 | **20298_73** |
| 4186_46 | 8380_74 | 20400_32 |
| 4320_27 | 8388_15 | *20548_19* |
| 4383_42 | 8520_42 | *20633_88* |
| 4546_80 | 8575_39 | *20810_86* |
| 4613_35 | 8575_6 | **21297_65** |
| 4665_62 | 8664_7 | 21510_19 |
| 4667_77 | 8794_9 | 22068_17 |
| 4668_42 | 8797_34 | **22237_35** |
| 4668_66 | 8836_15 | 23947_78 |
| **4668_81** | 8847_48 | 24442_62 |
| 4729_44 | 8849_28 | 25322_42 |
| 4832_89 | 9076_28 | **25380_52** |
| 4844_25 | 9076_54 | *25380_58* |
| *4847_58* | 9098_83 | **25380_84** |
| 4956_31 | 9169_48 | **25405_78** |
| 4970_90 | 9196_22 | **25627_51** |
| *4975_23* | 9196_23 | **25748_78** |
| **4975_68** | 9196_87 | 25881_17 |
| **4975_9** | 9275_68 | 25888_33 |
| 5045_48 | 9275_76 | 25888_38 |
| 5070_52 | 9275_79 | 25962_11 |
| 5084_54 | 9275_81 | **26519_27** |
| 5084_64 | 9508_72 | 26611_88 |
| 5161_82 | **9554_49** | *26726_84* |
| 5170_73 | 9881_90 | 18_69 |
| 5204_27 | 10070_67 | 1467_19 |
| 5247_15 | 10464_33 | 5791_60 |
| 5298_76 | 10464_52 | 7115_30 |
| 5354_34 | 10681_10 | *7115_35* |
| 5363_56 | **10832_84** | 10964_88 |
| 5394_86 | 10987_75 | 10964_89 |
| 5419_44 | 11131_89 | 10964_90 |
| 5420_31 | 11217_9 | 11110_18 |
| **5439_27** | 11315_68 | 18848_34 |
| 5443_24 | **11531_7** | 18848_38 |
| 5443_32 | 11634_72 | 19165_90 |
| 5443_6 | 11909_85 |  |
| 5443_67 | 11989_13 |  |
| *5515_42* | 12014_82 |  |
| 5522_80 | 12021_80 |  |
| 5807_51 | 12155_52 |  |
| 5842_16 | 12228_13 |  |
| 5978_53 | 12236_9 |  |
| 6026_30 | 12314_60 |  |
| 6026_53 | 12363_39 |  |
| 6186_46 | 12580_62 |  |
| 6190_47 | 12639_88 |  |
| 6191_10 | 12651_47 |  |
| 6203_48 | 12680_26 |  |
| 6228_45 | **12767_68** |  |
| 6228_84 | 12880_57 |  |
| 6236_60 | 12884_30 |  |
| 6288_14 | 13012_81 |  |
| 6346_68 | 13075_87 |  |
| 6491_67 | **13384_35** |  |
| 6579_25 | 13561_17 |  |
| 6591_67 | 13561_70 |  |
| 6604_68 | 13761_22 |  |
| 6645_6 | 13805_16 |  |
| 6688_49 | 13824_83 |  |
| 6759_11 | **13891_65** |  |
| 6805_6 | 14003_84 |  |
| 6810_7 | 14197_88 |  |
| 6810_79 | 14197_90 |  |
| 6887_79 | 14316_32 |  |
| 6888_15 | 14326_62 |  |
| 6888_38 | **14394_27** |  |
| 6945_90 | 14415_20 |  |
| 6976_68 | 14532_68 |  |
| 7042_35 | 14564_90 |  |
| 7114_22 | 15121_78 |  |
| 7120_90 | *15446_21* |  |
| *7203_45* | 15508_47 |  |
| 7318_31 | 15638_70 |  |
| 7343_38 | **15645_89** |  |
| 7361_21 | 15759_32 |  |
| 7376_36 | 15759_54 |  |
| 7408_7 | **15821_29** |  |
| 7423_59 | 15903_58 |  |
| 7423_71 | **16110_74** |  |
| 7540_65 | *16229_44* |  |
| 7626_32 | 16388_50 |  |
| 7637_73 | 16442_35 |  |
| 7648_39 | 16442_83 |  |
| 7700_76 | 16442_86 |  |
| 7724_27 | 16470_49 |  |
| 7724_45 | 16691_70 |  |
| 7737_36 | 16771_68 |  |
| 7846_50 | 16961_32 |  |
| 7857_24 | 17189_71 |  |
| 7857_32 | 17191_81 |  |
| 7857_74 | 17259_54 |  |
| 7857_8 | 17259_55 |  |
| 7882_64 | 17320_31 |  |
| 7941_58 | 17369_30 |  |
| 7997_36 | 17377_63 |  |
| 7999_74 | 17406_14 |  |
| 8325_27 | 17691_29 |  |
| 8380_74 | 17793_42 |  |
| 8520_42 | 17856_41 |  |
| 8540_90 | 17865_54 |  |
| 8563_90 | 17939_43 |  |
| 8575_39 | 18011_51 |  |
| 8575_6 | 18013_68 |  |
| 8635_12 | 18084_42 |  |
| 8655_19 | 18084_67 |  |
| 8735_68 | 18175_82 |  |
| 8741_86 | 18180_41 |  |
| *8782_41* | 18180_85 |  |
| 8794_56 | 18180_90 |  |
| 8794_9 | 18272_54 |  |
| 8797_34 | 18286_51 |  |
| 8797_89 | **18391_26** |  |
| 8836_15 | 18411_69 |  |
| 8847_48 | 18439_32 |  |
| 8849_28 | 18473_63 |  |
| 8922_62 | 18555_47 |  |
| 9076_28 | 18645_34 |  |
| 9076_31 | **18669_56** |  |
| 9076_54 | 18692_21 |  |
| 9098_83 | 18692_22 |  |
| 9163_31 | 18800_64 |  |
| 9169_48 | 18832_29 |  |
| 9196_22 | 18968_21 |  |
| 9196_23 | 19146_65 |  |
| 9196_87 | 19197_77 |  |
| 9203_74 | 19256_31 |  |
| 9211_22 | 19284_7 |  |
| 9275_68 | 19284_8 |  |
| 9275_76 | 19330_41 |  |
| 9275_79 | 19478_77 |  |
| 9275_81 | 19738_49 |  |
| 9281_51 | 19738_6 |  |
| 9387_50 | 19752_32 |  |
| 9387_7 | 19844_50 |  |
| 9431_43 | 19880_7 |  |
| 9431_86 | 20070_71 |  |
| 9471_77 | 20140_47 |  |
| 9508_72 | 20263_79 |  |
| **9554_49** | 20298_27 |  |
| 9653_60 | **20298_73** |  |
| *9976_51* | 20311_14 |  |
| 9996_36 | 20314_14 |  |
| 10070_67 | 20314_46 |  |
| 10141_55 | 20556_14 |  |
| 10156_85 | 20556_31 |  |
| 10159_36 | 20556_7 |  |
| 10361_56 | 20559_46 |  |
| *10366_19* | 20561_41 |  |
| 10464_33 | *20633_88* |  |
| 10464_52 | 20721_17 |  |
| 10485_37 | *20810_86* |  |
| *10498_47* | 20868_67 |  |
| 10527_56 | 20868_9 |  |
| 10529_34 | 20885_25 |  |
| 10587_84 | 20933_88 |  |
| 10681_10 | 21167_6 |  |
| 10810_32 | **21297_65** |  |
| *10832_30* | 21362_67 |  |
| *10832_46* | 21381_83 |  |
| *10832_74* | 21413_20 |  |
| **10832_84** | 21413_21 |  |
| 10873_33 | 21413_48 |  |
| 10917_59 | 21503_20 |  |
| *10987_36* | 21696_25 |  |
| 10987_74 | 22000_20 |  |
| 10987_75 | 22000_86 |  |
| 10987_8 | 22011_10 |  |
| 10993_65 | 22011_82 |  |
| 11126_44 | 22123_81 |  |
| 11131_89 | **22237_35** |  |
| 11217_9 | 23849_13 |  |
| 11265_39 | 24019_69 |  |
| 11315_68 | 24336_71 |  |
| 11407_80 | 24406_47 |  |
| 11431_85 | 24509_20 |  |
| **11531_7** | 24636_63 |  |
| 11634_72 | 24673_67 |  |
| 11696_20 | 24892_12 |  |
| 11696_43 | 24908_10 |  |
| 11748_62 | 25107_50 |  |
| 11784_88 | 25114_12 |  |
| 11810_49 | 25246_11 |  |
| 11909_85 | **25380_52** |  |
| 11966_39 | **25380_84** |  |
| 11989_13 | 25405_6 |  |
| 12014_82 | **25405_78** |  |
| 12031_71 | 25405_9 |  |
| 12041_65 | 25433_29 |  |
| 12155_52 | 25433_34 |  |
| 12228_13 | 25433_45 |  |
| 12236_9 | 25433_82 |  |
| 12314_60 | **25627_51** |  |
| 12363_39 | 25646_16 |  |
| 12370_76 | **25748_78** |  |
| 12370_81 | 25993_87 |  |
| 12377_67 | 26222_72 |  |
| 12419_58 | 26293_14 |  |
| 12580_62 | 26340_40 |  |
| 12639_88 | 26431_77 |  |
| 12651_47 | 26431_8 |  |
| 12651_75 | **26519_27** |  |
| 12674_8 | 26625_75 |  |
| 12680_26 | 26721_7 |  |
| **12767_68** | 26721_89 |  |
| 12880_57 | 26781_56 |  |
| 12884_30 | 26880_82 |  |
| 12936_75 | 26952_89 |  |
| 12969_14 | 27011_89 |  |
| 12969_15 |  |  |
| 12969_59 |  |  |
| 13012_81 |  |  |
| 13020_90 |  |  |
| 13075_87 |  |  |
| 13109_21 |  |  |
| 13197_42 |  |  |
| 13260_51 |  |  |
| 13270_21 |  |  |
| 13286_17 |  |  |
| 13303_49 |  |  |
| 13303_64 |  |  |
| 13303_79 |  |  |
| 13374_27 |  |  |
| **13384_35** |  |  |
| 13438_71 |  |  |
| 13586_43 |  |  |
| 13713_14 |  |  |
| 13727_6 |  |  |
| 13727_69 |  |  |
| 13744_54 |  |  |
| 13758_31 |  |  |
| 13824_83 |  |  |
| **13891_65** |  |  |
| 13966_68 |  |  |
| 13983_54 |  |  |
| 14018_57 |  |  |
| 14050_63 |  |  |
| 14076_10 |  |  |
| 14117_19 |  |  |
| 14117_7 |  |  |
| 14139_22 |  |  |
| 14139_68 |  |  |
| 14185_21 |  |  |
| 14197_86 |  |  |
| 14197_88 |  |  |
| 14197_90 |  |  |
| 14287_61 |  |  |
| 14310_37 |  |  |
| 14316_11 |  |  |
| 14316_32 |  |  |
| 14326_62 |  |  |
| **14394_27** |  |  |
| 14415_20 |  |  |
| 14421_56 |  |  |
| 14532_68 |  |  |
| 14557_41 |  |  |
| 14564_90 |  |  |
| *14571_64* |  |  |
| 14674_22 |  |  |
| 14967_65 |  |  |
| 15022_55 |  |  |
| 15090_47 |  |  |
| 15171_47 |  |  |
| 15203_19 |  |  |
| 15205_46 |  |  |
| 15205_67 |  |  |
| 15245_72 |  |  |
| 15261_65 |  |  |
| 15301_34 |  |  |
| 15459_62 |  |  |
| 15508_47 |  |  |
| 15638_54 |  |  |
| 15638_70 |  |  |
| **15645_89** |  |  |
| *15660_9* |  |  |
| 15687_33 |  |  |
| 15722_73 |  |  |
| 15759_32 |  |  |
| 15759_54 |  |  |
| **15821_29** |  |  |
| 15876_15 |  |  |
| 15900_50 |  |  |
| 15903_35 |  |  |
| 15903_58 |  |  |
| 15903_76 |  |  |
| 15903_78 |  |  |
| 16087_68 |  |  |
| **16110_74** |  |  |
| 16161_58 |  |  |
| 16161_6 |  |  |
| 16161_63 |  |  |
| 16161_64 |  |  |
| 16206_40 |  |  |
| 16232_32 |  |  |
| 16232_64 |  |  |
| 16300_20 |  |  |
| *16309_22* |  |  |
| 16339_61 |  |  |
| 16356_41 |  |  |
| 16388_50 |  |  |
| 16393_64 |  |  |
| 16396_57 |  |  |
| 16442_35 |  |  |
| 16442_83 |  |  |
| 16442_86 |  |  |
| 16466_34 |  |  |
| 16470_49 |  |  |
| 16517_82 |  |  |
| 16691_70 |  |  |
| 16771_68 |  |  |
| 16809_40 |  |  |
| 16837_37 |  |  |
| 16869_84 |  |  |
| 16891_32 |  |  |
| 16943_74 |  |  |
| 16961_32 |  |  |
| 17189_71 |  |  |
| 17191_81 |  |  |
| 17259_54 |  |  |
| 17259_55 |  |  |
| 17369_30 |  |  |
| 17377_63 |  |  |
| 17382_16 |  |  |
| 17406_14 |  |  |
| 17466_32 |  |  |
| 17480_65 |  |  |
| 17581_60 |  |  |
| 17581_65 |  |  |
| 17632_52 |  |  |
| 17691_29 |  |  |
| 17720_35 |  |  |
| 17731_31 |  |  |
| 17793_42 |  |  |
| 17819_48 |  |  |
| 17853_46 |  |  |
| 17865_54 |  |  |
| 17929_79 |  |  |
| 17929_9 |  |  |
| 17939_43 |  |  |
| 17998_24 |  |  |
| 18011_51 |  |  |
| 18013_68 |  |  |
| 18084_42 |  |  |
| 18084_67 |  |  |
| *18135_35* |  |  |
| 18175_82 |  |  |
| 18178_75 |  |  |
| 18180_41 |  |  |
| 18180_85 |  |  |
| 18180_90 |  |  |
| 18238_55 |  |  |
| 18238_56 |  |  |
| 18272_54 |  |  |
| 18286_51 |  |  |
| 18296_75 |  |  |
| **18391_26** |  |  |
| 18411_69 |  |  |
| 18439_32 |  |  |
| 18444_28 |  |  |
| 18473_63 |  |  |
| 18481_72 |  |  |
| 18555_47 |  |  |
| 18642_16 |  |  |
| **18669_56** |  |  |
| 18692_21 |  |  |
| 18692_22 |  |  |
| 18701_58 |  |  |
| 18701_60 |  |  |
| 18701_83 |  |  |
| 18800_64 |  |  |
| 18832_29 |  |  |
| 18865_67 |  |  |
| 18968_21 |  |  |
| 18972_74 |  |  |
| 19146_65 |  |  |
| 19197_77 |  |  |
| 19218_80 |  |  |
| 19252_8 |  |  |
| 19256_31 |  |  |
| 19284_24 |  |  |
| 19284_32 |  |  |
| 19284_7 |  |  |
| 19284_8 |  |  |
| 19302_50 |  |  |
| 19330_39 |  |  |
| 19353_39 |  |  |
| 19478_77 |  |  |
| 19487_24 |  |  |
| 19654_11 |  |  |
| 19738_49 |  |  |
| 19738_6 |  |  |
| 19752_32 |  |  |
| 19819_35 |  |  |
| 19844_50 |  |  |
| 19898_23 |  |  |
| 19898_6 |  |  |
| 19898_61 |  |  |
| 19960_23 |  |  |
| 20015_23 |  |  |
| 20047_71 |  |  |
| 20047_79 |  |  |
| 20047_87 |  |  |
| 20047_88 |  |  |
| 20062_71 |  |  |
| 20070_71 |  |  |
| 20106_11 |  |  |
| 20117_82 |  |  |
| 20140_47 |  |  |
| 20263_79 |  |  |
| 20298_27 |  |  |
| **20298_73** |  |  |
| 20314_14 |  |  |
| 20314_46 |  |  |
| 20527_29 |  |  |
| *20548_19* |  |  |
| 20556_14 |  |  |
| 20556_31 |  |  |
| 20556_59 |  |  |
| 20556_7 |  |  |
| 20559_46 |  |  |
| 20561_41 |  |  |
| 20596_34 |  |  |
| 20641_46 |  |  |
| 20721_17 |  |  |
| 20868_56 |  |  |
| 20868_67 |  |  |
| 20868_9 |  |  |
| 20885_25 |  |  |
| 20910_86 |  |  |
| 20929_40 |  |  |
| 20933_88 |  |  |
| 20942_64 |  |  |
| 20955_8 |  |  |
| 21028_28 |  |  |
| 21125_75 |  |  |
| 21138_65 |  |  |
| 21167_6 |  |  |
| **21297_65** |  |  |
| 21362_67 |  |  |
| 21381_83 |  |  |
| 21413_20 |  |  |
| 21413_21 |  |  |
| 21413_48 |  |  |
| 21418_49 |  |  |
| 21468_29 |  |  |
| 21468_89 |  |  |
| 21503_20 |  |  |
| 21600_62 |  |  |
| 21679_27 |  |  |
| 21696_25 |  |  |
| 21717_37 |  |  |
| 21746_21 |  |  |
| 21762_13 |  |  |
| 21853_20 |  |  |
| 22000_20 |  |  |
| 22000_86 |  |  |
| 22011_10 |  |  |
| 22011_82 |  |  |
| 22100_34 |  |  |
| 22122_62 |  |  |
| 22123_81 |  |  |
| **22237_35** |  |  |
| 23183_26 |  |  |
| 23296_73 |  |  |
| 23306_43 |  |  |
| 23346_81 |  |  |
| 23361_35 |  |  |
| 23535_28 |  |  |
| 23849_13 |  |  |
| 23969_73 |  |  |
| 24019_69 |  |  |
| 24230_82 |  |  |
| 24257_63 |  |  |
| 24336_71 |  |  |
| 24373_14 |  |  |
| 24380_74 |  |  |
| 24384_24 |  |  |
| 24406_47 |  |  |
| 24509_20 |  |  |
| 24587_47 |  |  |
| 24636_63 |  |  |
| 24673_67 |  |  |
| 24804_65 |  |  |
| 24882_77 |  |  |
| 24892_12 |  |  |
| 24892_33 |  |  |
| 24908_10 |  |  |
| 24939_35 |  |  |
| 24949_72 |  |  |
| 24954_38 |  |  |
| 25064_55 |  |  |
| 25067_48 |  |  |
| 25107_50 |  |  |
| 25246_11 |  |  |
| 25310_43 |  |  |
| 25310_48 |  |  |
| 25366_26 |  |  |
| **25380_52** |  |  |
| *25380_58* |  |  |
| **25380_84** |  |  |
| 25405_6 |  |  |
| **25405_78** |  |  |
| 25405_86 |  |  |
| 25405_9 |  |  |
| 25433_29 |  |  |
| 25433_34 |  |  |
| 25433_45 |  |  |
| 25433_82 |  |  |
| 25464_47 |  |  |
| 25562_55 |  |  |
| 25580_62 |  |  |
| 25599_88 |  |  |
| **25627_51** |  |  |
| 25630_84 |  |  |
| 25652_55 |  |  |
| **25748_78** |  |  |
| 25993_87 |  |  |
| 26091_42 |  |  |
| 26091_9 |  |  |
| 26141_15 |  |  |
| 26141_84 |  |  |
| 26222_72 |  |  |
| 26293_14 |  |  |
| 26293_68 |  |  |
| 26312_54 |  |  |
| 26337_72 |  |  |
| 26340_40 |  |  |
| 26371_65 |  |  |
| 26391_35 |  |  |
| 26391_71 |  |  |
| 26431_77 |  |  |
| 26431_8 |  |  |
| **26519_27** |  |  |
| 26550_72 |  |  |
| 26570_14 |  |  |
| 26625_75 |  |  |
| 26721_7 |  |  |
| 26721_89 |  |  |
| *26726_84* |  |  |
| 26781_56 |  |  |
| 26880_82 |  |  |
| 26937_69 |  |  |
| 26952_89 |  |  |
| 27011_89 |  |  |
| 27045_28 |  |  |
